# Bioinspired Multifunctional Glass Surfaces through Regenerative Secondary Mask Lithography

**DOI:** 10.1002/adma.202102175

**Published:** 2021-09-13

**Authors:** Martyna Michalska, Sophia K. Laney, Tao Li, Mark Portnoi, Nicola Mordan, Elaine Allan, Manish K. Tiwari, Ivan P. Parkin, Ioannis Papakonstantinou

**Affiliations:** ^1^ Photonic Innovations Lab Department of Electronic & Electrical Engineering University College London Torrington Place London WC1E 7JE UK; ^2^ Division of Biomaterials and Tissue Engineering UCL Eastman Dental Institute Royal Free Campus University College London Pond Street London NW3 2QG UK; ^3^ Department of Microbial Diseases UCL Eastman Dental Institute Royal Free Campus University College London Rowland Hill Street London NW3 2PF UK; ^4^ Nanoengineered Systems Laboratory Department of Mechanical Engineering University College London Torrington Place London WC1E 7JE UK; ^5^ Wellcome/EPSRC Centre for Interventional and Surgical Sciences (WEISS) University College London London W1W 7TS UK; ^6^ Department of Chemistry University College London 20 Gordon Street London WC1H 0AJ UK

**Keywords:** antimicrobials, antireflection, bioinspired multifunctionality, glass, nanopatterning, periodic nanoarrays, self‐cleaning

## Abstract

Nature‐inspired nanopatterning offers exciting multifunctionality spanning antireflectance and the ability to repel water/fog, oils, and bacteria; strongly dependent upon nanofeature size and morphology. However, such patterning in glass is notoriously difficult, paradoxically, due to the same outstanding chemical and thermal stability that make glass so attractive. Here, regenerative secondary mask lithography is introduced and exploited to enable customized glass nanopillars through dynamic nanoscale tunability of the side‐wall profile and aspect ratio (>7). The method is simple and versatile, comprising just two steps. First, sub‐wavelength scalable soft etch masks (55–350 nm) are generated through an example of block copolymer micelles or nanoimprinted photoresist. Second, their inherent durability problem is addressed by an innovative cyclic etching, when the original mask becomes embedded within a protective secondary organic mask, which is tuned and regenerated, permitting dynamic nanofeature profiling with etching selectivity >1:32. It is envisioned that such structuring in glass will facilitate fundamental studies and be useful for numerous practical applications—from displays to architectural windows. To showcase the potential, glass features are tailored to achieve excellent broadband omnidirectional antireflectivity, self‐cleaning, and unique antibacterial activity toward *Staphylococcus aureus*.

## Graphical abstract

A difficulty of bioinspired nanostructuring in glass is addressed by regenerative secondary mask lithography, a facile and versatile fabrication concept utilizing dynamic nanoscale tunability of the side‐wall profile and aspect ratio, leading to customized nanoarrays. Such nanopatterning affords antireflectance and the ability to repel water/fog and bacteria, highly desired in many applications—from displays to automotive and architectural windows.

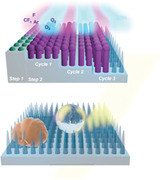

## 1 Introduction

Nature has evolved smart surfaces by organizing ordinary building blocks into sub‐wavelength patterns to impart extraordinary properties, often of multifunctional character.^[^ ^1^, ^2^, ^3^ ^]^ In this regard, bioinspired nanopatterning with purpose‐tailored geometries (typically high aspect ratio > 1) has become a fast‐evolving field, underpinning fundamental research and enabling novel applications from antireflective surfaces,^[^ ^4^, ^5^ ^]^ photonic membranes,^[^ ^6^ ^]^ biological metamaterials,^[^ ^7^ ^]^ de‐icing^[^ ^8^ ^]^ and dew‐repelling coatings,^[^ ^9^ ^]^ to mechano‐bactericidal strategies.^[^ ^10^ ^]^ Broadly, this multifunctionality is inherent to and bridged by the nanocone structure, yet such patterning in glass (SiO_2_) remains a bottleneck due to its high chemical stability alongside structuring at the nanoscale itself, which becomes increasingly challenging to manage as the pattern resolution advances (pitch < 100 nm). Attaining control over the pitch has, however, transpired to be necessary to advance existing, or unlock additional functionality; with theoretical models and experimental studies in silicon indicating that at these smaller length scales, nanocones are not only capable of resisting droplet impacts of higher velocity, but also of repelling water at the microscale—answering the need for antifogging surfaces.^[^ ^9^ ^]^ Practically, this enables self‐cleaning under various weather conditions permitting raindrops or dew to manage pollutants, for instance. Critical to the performance are also the profile and aspect ratio, where higher aspect ratio ensures smoother refractive index gradients, hence better antireflectance,^[^ ^11^ ^]^ as well as it impacts flexibility of the cone, useful in boosting antimicrobial activity (e.g., aspect ratio ≈ 10).^[^ ^12^ ^]^ The latter functionality, widely demonstrated in other materials,^[^ ^10^ ^]^ remains yet to be explored in glass; likely, due to the aforementioned structuring challenges.

Currently, such nanopatterning in glass is largely managed by multistep photolithographic processes^[^ ^13^ ^]^ or somewhat simplified (de‐wetting; mask‐less) but at the expense of feature fine‐control,^[^ ^14^, ^15^, ^16^ ^]^ necessary to tune a combination of the properties. Additionally, despite pushing the resolution limits by deep‐ultraviolet lithography or multiple patterning,^[^ ^13^, ^17^ ^]^ these complex strategies and costly equipment make such routes less appealing for mass‐production when compared to self‐assembling or imprinting methods. For example, block copolymers (BCPs) show a high‐degree order at 10–100 nm scales (perfectly matching bio‐patterns) and uniformity (m^2^), while being economic and compatible with semiconductor technologies.^[^ ^18^ ^]^ Similarly, nanoimprint lithography (NIL) generates high‐resolution etch masks in photoresists (down to tens nm), and can be scaled up into a continuous roll‐to‐roll process.^[^ ^19^ ^]^ Ultimately, the material/device performance depends on the quality and aspect ratio of the pattern transferred into the glass via etching, which in turn depends on the mask's durability. To this end, the rapid BCP consumption as a soft mask alongside its limited height (tens nm), have been mitigated through a use of organic‐organometallic BCPs^[^ ^20^ ^]^ or sequential infiltration synthesis;^[^ ^21^ ^]^ by forming a hard mask via metal incorporation into a BCP block. This enables nanopillars with aspect ratio ≈ 5.^[^ ^22^ ^]^ A concept variation utilizing iron salt^[^ ^23^ ^]^ or gold nanoparticles^[^ ^24^ ^]^ led to the only reported aspect ratio ≈ 20, however with disordered patterns above ≈4.^[^ ^11^ ^]^ Unlike BCP, a thicker photoresist mask can be imprinted (or nanospheres employed) to compensate the degradation issue. Yet at such scales, electrostatic attraction between adjacent mask sections leads to buckling, beyond certain mask thickness, hindering significant improvements.^[^ ^25^ ^]^ Whilst overall metal masks improve etching selectivity, they introduce an additional step, cost, and potential source of contamination (diffusion into the substrate, non‐volatile byproducts/debris deposition).^[^ ^26^ ^]^ Evidently, with no other perceivable means to enhance the existing, but limited glass etching chemistry (fluorocarbon‐based plasma accompanied by the formation of a carbopolymer etching‐inhibitor layer), these mitigation routes were required and remain the state‐of‐the‐art in soft mask‐mediated glass nanofabrication. As such, developing a strategy to prevent soft mask degradation, compatible with scalable masking techniques, whilst erasing the need for soft‐to‐hard‐mask conversion or mask thickening (limited for BCPs), represents a major materials and nanofabrication challenge.

**Figure 1:**
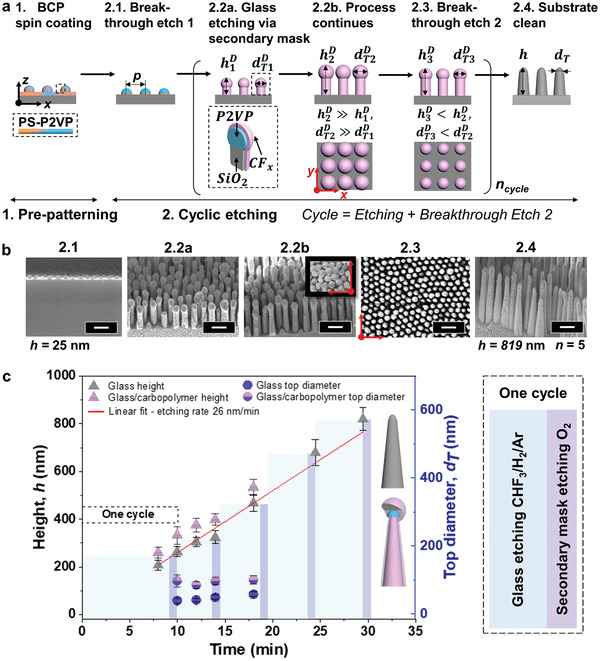
Two‐step fabrication process of glass nanopillars. a) Schematic illustration of regenerative secondary mask lithography consisting of: (Step 1) Surface pre‐patterning using block copolymer (BCP) lithography, and (Step 2) cyclic reactive ion etching (RIE). Left to right: 1) Direct spin‐coating of pre‐assembled BCP micelles [micelle = P2VP core (blue) and PS matrix (orange)]. The second step proceeds entirely within the RIE chamber but is broken down here into sub‐steps: 2.1) Breakthrough etch 1 using O_2_ plasma to remove PS matrix; 2.2a,b) Anisotropic etching of glass (gray) during time *t* = *a* or *b* (*b*> *a*), using CHF_3_/H_2_/Ar plasma, with simultaneous carbopolymer deposition (CF*_x_*; pink) forming a secondary mask. The evolving pillars at time *t* = *a*, possess height $h_{1}^{D}$ and top diameter $d_{\text{T1}}^{D}$ (where superscript *D* refers to a structure with secondary mask), which both increase with the etching time until a maximum is reached (limited by $d_{T}^{D}$) at time *t* = *b*; (2.3) Breakthrough etch 2 using O_2_ plasma to refine the secondary mask; (2.4) Further etching with O_2_ yields clean glass nanoarray. Steps (2.2–2.3) can be cycled *n* times to elicit structures of given *h*. The dashed lines indicate cross sections. b) Corresponding SEM images to the schematics (2.1–2.4), tilted 45° and top views. Five cycles result in glass pillars of *h* = 819 nm. Scale bars: 200 nm. c) An example of nanostructure evolution during *n* = 5 cycles, monitored by changes in height and top diameter, with and without deposited carbopolymer.

Herein, we address this challenge and present facile templating of glass nanostructures (pillars/cones) of varying aspect ratio directly from the organic morphology (on the example of BCP and photoresist), with high selectivity (>1:32). At its core, our approach comprises just two steps (i) soft mask pre‐patterning and (ii) our innovative cycling etching. We utilize H_2_ addition into the glass etching chemistry (CHF_3_/Ar) to trigger secondary (protective) mask formation around the original mask through well‐controlled carbopolymer deposition. Importantly, the secondary mask is dynamic, as it can be reduced and regenerated, allowing side‐wall profiling and multiple etching cycles (**Figure** **1a**). We call this original mechanism regenerative secondary mask lithography (RSML), which represents a generic solution for enhancing etching selectivity to elicit deep structures templated from thin soft masks, and can be applied to double‐sided patterning. Finally, we achieve nanostructured glass with high transparency, broadband, haze‐free, omnidirectional antireflectivity (>97.5% transmission calibrated to human eye response); impact‐resistant superhydrophobicity (tested up to 4.4 m s^−1^); and lastly, the first demonstrated antibacterial properties on a glass surface toward *Staphylococcus aureus* with a competitive >81% killing efficiency.

## 2 Results and Discussion

### 2.1 Two‐Step Fabrication—Regenerative Soft Mask Lithography (RSML)

Figure 1a,b and Video S1 (Supporting Information) show schematically the RSML process with corresponding scanning electron microscopy (SEM) images for an exemplary *p* = 110 nm. It begins with surface pre‐patterning. Here, pre‐assembled block copolymer micelles comprised of PS‐*b*‐P2VP [poly(styrene‐*block*‐2vinylpyridine)] are spin‐coated onto the substrate forming hexagonally packed micellar bumps under ambient conditions [Figure 1a‐1; Section S1, Supporting Information].^[^ ^27^ ^]^ Broadly, the phase segregation of the blocks is driven thermodynamically (molecular weight *M*_w_, block ratio, composition, and degree of the blocks immiscibility) and kinetically (vapor pressure, humidity), providing a multiparameter space to accommodate specific applications.^[^ ^18^, ^28^ ^]^ We use a combination of *M*_w,_ concentration, solvent, and spin speed to generate patterns with *p* = 55–300 nm of unimodal or bimodal distributions; the latter enables binary or hierarchical structures, for instance (Figure S1, Supporting Information).

The second step, aiming at pattern transfer into the substrate, proceeds in a single reactive ion etching (RIE) process. For understanding, it is broken down into sub‐steps (Steps 2.1–4; Figure 1a), where oxygen plasma is first used as a breakthrough etch (2.1) to define mask diameter and uncover the underlying material to be etched (Figure S2, Supporting Information). Subsequently, we use fluorohydrocarbon plasma (CHF_3_/Ar) to selectively etch SiO_2_. The modest F/C ratio prompts moderate etching rates, critical for controlling etching profiles of narrow high‐aspect‐ratio nanostructures (Section S2 and Figure S3, Supporting Information). We add hydrogen (H_2_) to the reaction, harnessing its ability to scavenge fluorine to form HF (aiding glass etching), and meanwhile lower the F/C ratio, thereby providing fluorocarbon species (CF*_x_*) of high sticking probability.^[^ ^29^ ^]^ Under such conditions, fluorocarbon deposition (polymerization), fluorocarbon etching (surface reaction) and ion‐assisted glass etching occur simultaneously. We take advantage of this shift in plasma character, from etching to more polymerizing, and establish the conditions which induce secondary mask formation around the BCP originating from the CF*_x_*, inhibiting its consumption (Step 2.2a). Buildup control is obtained by adjusting the energy of Ar ions (vide infra). This etching advancement alone yields only moderate heights (≈300 nm) as the growing mask becomes an obstacle in deeper etching, particularly for such high‐resolution patterns (2.2b). Importantly, we find that it can be controllably reduced under a brief and mild oxygen plasma (Step 2.3—breakthrough etch 2), and can crucially regenerate in the following glass etching (discussed later).

To achieve high‐aspect‐ratio nanostructures, this cycle (etching and breakthrough 2) needs to be repeated and optimized such that etching, deposition, and Ar ion sputtering rates (*R* ^E^, *R* ^D^, *R* ^S^, respectively) are balanced. Finally, complete CF*_x_* removal (2.4) is achieved by applying a harsher oxygen plasma. Note in Figure 1b, how the initial organic mask with height *h*_BCP_ = 25 nm yields glass nanopillars with impressive *h* > 800 nm (five cycles), indicating the etching selectivity exceeds 1:32. Graphical representation of the heights and top diameters of the evolving structures with and without the deposited layer (*h* ^D^, *h*, $d_{T}^{D}$, *d*_T_, respectively), quantitatively expresses, and further conceptualizes the process (Figure 1c). The BCP mask contributes to both *h* ^D^ and $d_{T}^{D}$, and by assuming it is intact throughout, we can derive the deposition thickness at time *t* as

(1) $$\Delta h=h_{t}^{D}-(h_{t}+h_{\text{BCP}})$$

and

(2) $$\Delta d_{T}=d_{T,t}^{D}-d_{\text{BCP}}$$

To induce the secondary mask, *R* ^D^ > *R* ^S^ is required, yet for high profile anisotropy this difference should be modest (in this example, *R* ^D^ = 4 ± 1 nm min^−1^), to ensure sufficient deposition protecting the BCP whilst enough physical bombardment to permit anisotropy (∝Ar flow). After the breakthrough etch, which reduces the secondary mask (and thus $d_{T}^{D}$ and *h* ^D^), it becomes evident that during the subsequent etch the mask is regenerated, verified by a constant value at the end of each etch with average Δ*d*_T_ = 49 ± 2 nm and Δ*h* = 45 ± 4 nm.

**Figure 2:**
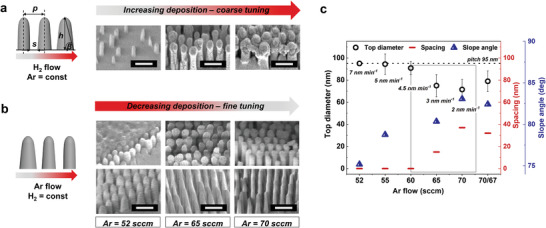
Manipulation of etching/deposition gas composition for anisotropy control. a,b) Schematics with corresponding SEM images (45° tilt) showing major trends in tuning glass etching profiles—through sidewall angle β (tapering)—templated from BCPs when using CHF_3_/H_2_/Ar plasma chemistry under varied H_2_ and Ar flows, respectively. The pillar/cone profiles (height *h*, pitch *p*, and spacing *s*) are correlated with the degree of CF*_x_* deposition (expressed as deposition rate *R* ^D^), which results in the secondary mask formation of diameter $d_{T}^{D}$ visualized by corresponding SEM images presenting glass nanopillars with *p* = 95 nm. a) Increasing H_2_ flow, results in thickening of the secondary mask around the BCP, leading to a decrease in β and premature etch stop. Conversely, for (b), increasing Ar flow a stronger physical bombardment occurs resulting in smaller deposition and larger β which is seen more clearly after removal of the mask (bottom row of SEM images). We establish the process conditions by first testing H_2_ flows until deposition occurs (coarse tuning). Second, Ar flow is used to fine‐tune *R* ^D^ and hence the profile, presented quantitatively by (c). c) Plot of $d_{T}^{D}$, *s*, and β versus Ar flow. Overall, the closer β is to 90°, the more anisotropic the profile, and the maximum $d_{T}^{D}$ is equivalent to the pitch (dotted line). The optimal process conditions are framed. Note, varying Ar during the process allows for further tuning resulting in base widening, for instance (70* = 70 sccm followed by 67 sccm).

To explain this etching scenario, we detail in **Figure** **2** how to induce and manage the secondary mask which ultimately controls the profile anisotropy/tapering (yielding vertical pillars/tapered cones, respectively)—characterized by the slope angle β (see Figure 2a). When a substantial amount of H_2_ is added (CHF_3_:H_2_≤ 3.3), mushroom‐like structures are formed around the mask at the top of the evolving nanostructures. At first glance, it seems surprising that the augmented deposition is formed solely around the mask. However, when ions hit the horizontal surfaces between the features, chemical reactions are induced by breaking the Si—O bonds. As opposed to the BCP, silicon reacts with fluorine while liberated oxygen burns the carbopolymer away as CO*_x_*. This indicates the mechanism to be relatively generic and applicable to other organic masks which we discuss later. Careful choosing of the CHF_3_:H_2_ ratio is required as larger ratio decreases the deposition so that the BCP degrades quickly, distorting the pattern (Figure 2a). Conversely, too small a ratio (≈2), augments the deposition, thus inhibiting etching. We attempt to alternate these two processes (etching‐ or deposition‐driven) to create a pseudo‐Bosch switching route, being successful in silicon and providing some advances in glass etching, e.g., advanced cyclic etching method.^[^ ^30^, ^31^ ^]^ Despite initial successes (Figure S4, Supporting Information; *p* ≈ 200 nm), we find frequent variations of H_2_ content and thus reaction chemistries difficult to manage, particularly at reduced pitch (≈100 nm).

Instead, we anticipate that varying an inert gas such as Ar at fixed H_2_ flow should affect ion bombardment and therefore when well‐balanced, an *R* ^S^ can be obtained that is a fraction slower that *R* ^D^. This enables more anisotropic etching through downward ion channeling off the sidewalls, hence imparting higher *R* ^E^ at the base allowing high‐aspect‐ratio nanostructures to be formed within non‐switching process.^[^ ^32^ ^]^ Indeed, we successfully control the secondary mask formation through precise adjustment of Ar flow (Figure 2b,c; Figure S5, Supporting Information) to provide control over β—which in turn dictates the nanostructures height at certain pitch (etch stop), and can affect the mechanical stability.^[^ ^33^ ^]^ We further quantify that approximately 2 ≤ *R* ^D^< 5 nm min^−1^ is optimal to form β > 75°. Whilst the profile significantly changes within the tested range, the *R* ^E^ is unaffected (Figure S6, Supporting Information).

**Figure 3:**
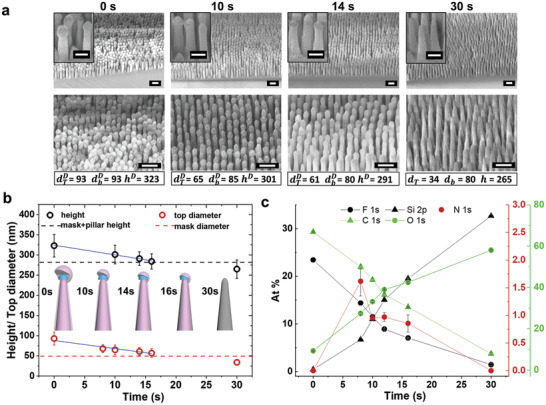
Effect of secondary mask tuning. a) Temporal evolution of reducing carbopolymer under mild O_2_ plasma, SEM images tilted at 45^o^. Height, top, and base diameters are determined ($h^{D}$, $d_{T}^{D}$, and $d_{b}^{D}$, respectively). b) Polymer reduction rate determined by plotting $h^{D}$ and $d_{T}^{D}$ as a function of O_2_ etching time. The lateral and vertical rates were determined by linear fits and are 2.0 ± 0.4 and 2.4 ± 0.1 nm s^−1^, respectively, hence indicating an isotropic etching. The rendered structures represent the changes in carbopolymer thickness where gray is a glass pillar, blue is the BCP (both blocks) mask, and pink is the deposited layer. c) Surface composition obtained from the survey spectra by X‐ray photoelectron spectroscopy (XPS) for the series of the samples treated with varying O_2_ etching time.

Having reached a point where the $d_{T}^{D}$ is approaching the pitch (due to *R* ^D^ >*R* ^S^), we introduce a brief oxygen etch which enables diameter control through reduction of the secondary mask (**Figure** **3**), hence permitting deeper etching. By knowing the O_2_ *R* ^E^, $d_{T}^{D}$, and *d*_BCP_, one can determine the necessary time for this breakthrough etch (Step 2.3) as shown in Figure 3b (inadequate = less space for further etching, excessive = mask degradation). Importantly, mild conditions are required to prevent mask distortion. The surface composition analysis obtained by XPS (Figure 3c; Figure S7, Supporting Information) further confirms that initially (*t* = 0 s; end of Step 2.2), only carbon and fluorine are present, in contrary to *t* = 30 s where silicon and oxygen make up >90% of the entire composition (Step 2.4). Interestingly, a trace amount of nitrogen appears (≈1%) throughout, originating from pyridine (P2VP), which is absent at the end of the etching; providing evidence the BCP is embedded within the CF*_x_* structure. Note, the attainable pattern quality across a surface as shown in Figure S8 in the Supporting Information and exemplary nanostructures with aspect ratio of 6 at *p*< 100 nm.

The great advantage of RSML‐mediated glass nanostructuring is its straightforward nature. This enables flexibility and facile optimization to accommodate various masks and targeted topographies of certain pitch, aspect ratio, and feature shape (vertical/tapered sidewalls and round/sharp apex)—principal attributes to manage photons, water/oils, and/or cells. We apply the established guidelines to BCP P400 (*p* = 260 nm) and yield glass nanopillars with *h*> 1 µm (Figures S9–S11, Supporting Information), which further validates the approach. Additionally, we etch glass using a photoresist mask (Section S7 and Figures S12–S13, Supporting Information) proving RSML is a generic solution to overcome soft mask degradation. Probing the mechanical stability of our attained surfaces, we performed the tape‐peel test on surfaces of lower and higher aspect ratio (AR = 2.5 and 5.5). Imaging of the surfaces via SEM afterward, revealed that the pillars survived the tape‐peel test (Figure S14, Supporting Information).

### 2.2 Application of Nanostructure Arrays

Through RSML, we now demonstrate the potential and quality of attainable topographies, particularly at reduced pitch (<100 nm), to accomplish high‐performance multifunctionality including antireflectivity, high‐transparency, superhydrophobicity, and antibacterial activity. We first optimize the nanocones to provide broadband antireflectivity over wide angles. Nanocones operate by adiabatically bridging the refractive index of the substrate with that of air (Figure S15a, Supporting Information). Pitch and height are dimensions known to play key roles in discerning the minimum and maximum wavelengths for which reflectance is suppressed.^[^ ^34^ ^]^ Through our model (Section S9, Supporting Information), a criterion of aspect ratio >2 is defined to effectively suppress reflectance across the visible range (Figure S15c, Supporting Information). Note, that if antireflection properties were to be maintained into the NIR, an aspect ratio of ≈5 would be required (Figure S15d, Supporting Information), demonstrating the need for high‐aspect‐ratio structures.

**Figure 4:**
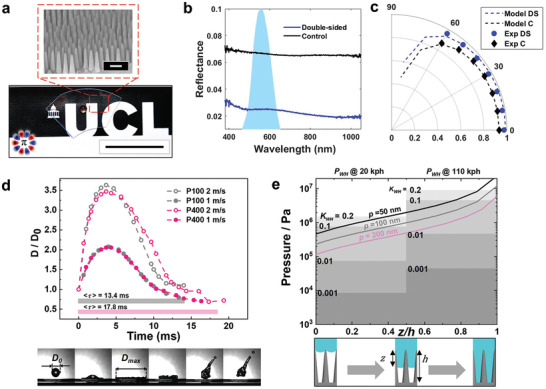
Optical properties and wettability characteristics. a) Photograph and SEM image of a double‐sided etched superhydrophobic glass after fluorosilane‐coating (note the round shape of a water droplet). Scale bars: 200 nm (SEM image) and 1 inch. b) Measured reflectance as a function of wavelength for flat quartz glass (control C; black) and double‐sided nanocone sample (DS; blue). The photopic response of the human eye is shown with light blue shaded area. c) Measured and calculated transmission of C and DS samples as a function of incident angle for incoherent, unpolarized light, for photopic calibrated data. d) Top: Sequential images of a droplet impacting a surface with an initial diameter *D*_0_ = 2.7 mm, expanding to a maximum diameter *D*_max_ = 9.45 mm as it spreads on the surface, followed by retraction and take‐off. The corresponding plot of drop diameter versus time with the *y*‐axis normalized to *D*_0_ is shown for P100 (gray) and P400 (pink) at two impacting velocities: 1.0 m s^−1^ (dots) and 2.0 m s^−1^ (circles). The average contact time <τ> of the droplet with the surface is indicated to be 13.4 ms and 17.8 ms for P100 and P400, respectively. e) Calculated capillary pressure *P*_c_ plotted for nanocones of pitch 50, 100, and 200 nm, as a function of the penetration percentage (*z*/*h*), where *z* is the depth of meniscus penetration and *h* is the total height. The water hammer pressure generated at impacts of 20 and 110 kph are marked on the graph, with varying values for the water hammer pressure coefficient *K*_WH_. The bottom row of schematics serves as a representation of the penetration depth *z*/*h*.

Every substrate has two interfaces (air‐glass and glass‐air) where abrupt refractive index changes occur, therefore we perform double‐sided patterning; a non‐trivial fabrication challenge, for which we develop a new process (Section S10 and Figure S16, Supporting Information). The reflectance of our double‐sided sample with *p* = 95 nm, aspect ratio ≈ 4 (**Figure** **4**a), is measured as a function of wavelength and compared against the control (flat) substrate (Figure 4b). Evidently, the reflectance of the sample (≈2.5%; calibrated against human photopic vision)^[^ ^35^ ^]^ is considerably lower than the control (≈6.8%) across the whole investigated spectral range. Notably for the chosen aspect ratio, reflectance is sustained to <2% in the near infrared (NIR) wavelengths up to 1100 nm (experimental limit), additionally unlocking NIR applications.^[^ ^11^ ^]^ Importantly, the resulting samples are haze‐free, as demonstrated in Figure S17 in the Supporting Information. We measure the transmittance as a function of the incident angle (Figure 4c; see the Experimental Section for details) and observe consistent antireflectivity up to 60°, establishing the robust broadband characteristics.^[^ ^11^, ^13^, ^34^, ^36^, ^37^, ^38^, ^39^ ^]^

Similarly to the optical requirements, engineering superhydrophobic nanocones with enhanced impact‐resistance, is achieved through: small pitch, tapered geometry, and uniform structuring.^[^ ^13^ ^]^ Crucial for preventing contact‐line pinning, a minimized solid fraction is obtained through brief post‐processing with diluted hydrofluoric acid (Figure S18, Supporting Information). Accordingly, we observe a substantial increase in advancing water contact angle (154–164°), and decrease in hysteresis (10–3°) for antireflective P100—rendered superhydrophobic (Figure S18 and Videos S2 and S3, Supporting Information). An additional indication of the global hydrophobicity is provided by ε,^[^ ^40^ ^]^ the restitution coefficient of a bouncing water droplet on a surface. We observe a remarkably high ε = 0.9 translating to 17 bounces (Figure S19 and Video S4, Supporting Information).

To investigate high‐speed impacts, we deposit droplets of increasing velocity *V* = 0.4–4.4 m s^−1^ at room temperature and observe whether pinning occurs; at *V* = 4.4 m s^−1^, P100 demonstrates no pinning (Video S5, Supporting Information). In a representative splashing sequence (Figure 4d), a droplet of *V* = 2 m s^−1^ contacts P100, spreading to a maximum diameter *D*_max_. Comparison of droplet diameter *D* as a function of time for two samples varying in pitch, P100 and P400 (*p* = 257 nm), highlights no difference in the expansion process, and shows consistency relating to higher velocity impacts (greater deformation). During the retraction process—driven by the minimization of droplet surface area and solid‐liquid contact—a shorter retraction time τ on P100 is observed, in agreement with previous reports relating to partial impalement.^[^ ^41^ ^]^ Whilst the higher density of P100 nanostructures suggests a greater wetted area than P400, the reduced pitch yields a higher resistive capillary pressure *P*_c_ (which also increases with penetration depth *z* owing to the tapered profile). The degree of meniscus penetration *z*/*h* can be estimated through comparison of *P*_c_ with the water hammer pressure *P*_WH_ generated by an impacting droplet. *P*_WH_ is an empirical parameter, proportional to the water hammer pressure coefficient *K*_WH_, which was found to largely vary with surface texture (0.001–0.2).^[^ ^41^, ^42^ ^]^ When considering *K*_WH_= 0.2 (generating the highest *P*_WH_), the structures indeed should withstand an impact of *V* = 2.0 m s^−1^ so that *z*/*h* for P100 is ≈30%, whereas for P400 it is ≈60% (Section S10, Supporting Information).^[^ ^43^ ^]^ The significantly lower infiltration for P100 highlights the need for such resolution. In Figure 4e, the trend becomes even more apparent when comparing the *P*_c_ for nanostructures of reducing pitch (*p* = 200–50 nm) to the *P*_WH_ (with *K*_WH_ varying between 0.001 and 0.2) generated at speeds of 20 and 110 kph; commonly found in practical settings. Furthermore, nanocones of increasing aspect ratio are also calculated to withstand higher droplet impacts owing to the greater unfavorable contact experienced between the droplet and the functionalized nanocones for an aspect ratio of 5 (for instance) compared to an aspect ratio of 1 at the same penetration depth (Figure S20, Supporting Information).

Clearly, reduced pitch benefits the discussed functionalities, however predicting antibacterial properties is more complex due to the multitude of contributing factors including both material (aspect ratio/elasticity/shape/pitch)^[^ ^44^ ^]^ and cellular features (rigidity/motility).^[^ ^45^ ^]^ The non‐trivial simulations have led to models which propose often opposite design criteria, with ambiguity concerning pitch but general agreement on the merits of sharper tips.^[^ ^46^ ^]^ Here, we focus on the interactions with *S. aureus* (Gram‐positive—highly rigid and thus harder to inactivate than Gram‐negative species),^[^ ^46^ ^]^ and for guidance, calculate the pressure exerted on cells by various topographies (Figures S21 and S22, Supporting Information).^[^ ^47^ ^]^ The data confirm a lower tip diameter generates higher pressure exerted on the cell, with pitch being inversely proportional (Figure S22, Supporting Information). For our P100 structures, (*p* = 110 nm; *d*_T_ = 21 nm), the model predicts a pressure of ≈10 MPa indicating that creep deformation can occur with the potential to rupture given sufficient nanopillar height.

**Figure 5:**
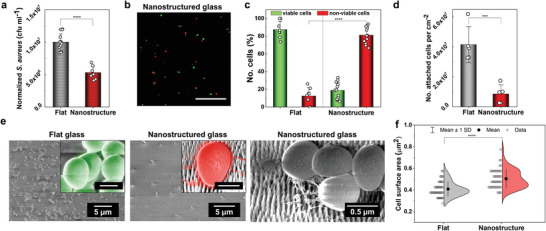
Antibacterial properties of glass nanostructures. a) Number of viable *S. aureus* cells after interacting with flat (control) and nanostructured glass surface (nanopillars *p* = 110 nm, *h* = 230 nm, *d*_t_ = 20 nm) in PBS for 18 h, assessed by viable counting. Nanostructures show a significant reduction in the number of viable bacteria with respect to the control (**** *P*< 0.0001, *t*‐test, the error bars show SD calculated from *n* = 3 independent experiments containing replicates ≥3; circular markers, the data were normalized to 1.0 × 10^7^ colony forming units per mL). b) Fluorescence microscopy images of *S. aureus* on nanostructured glass stained with LIVE/DEAD BacLight kit, where red dye indicates cells with compromised membranes (damaged/non‐viable) and green dye indicates viable cells. Scale bar: 20 µm. c) Plot showing antibacterial efficiency (81%, compared to 12% on the control) of the nanostructured glass expressed as a percentage of non‐viable cells out of the total counted cells (**** *P*< 0.0001, *t*‐test, error bars show SD, *n* = 3 independent experiments). d–f) SEM imaging reveals marked differences in the number of attached bacteria to the flat and nanostructured surface (*** *P*< 0.001, *t*‐test) (d) and in the morphology of attached cells (e), as can be seen in the SEM images, which is quantitively determined by an increase in the average projected cell surface area (**** *P*< 0.0001, *t*‐test) (f). SEM images are representative of three independent surfaces. Scale bars in insets: 0.5 µm.

Experimentally, we investigate the antibacterial properties by viable counting and surface fluorescent imaging, where live and damaged/dead cells are visualized based on their membrane integrity (**Figure** **5a**–c). Both measurements indicate significantly reduced numbers of viable bacteria after interacting with the nanostructures comparing to the control. The average proportion of non‐viable cells on the surface is 81%, matching our theoretical predictions and previous results obtained with silicon (83–85%)^[^ ^12^ ^]^—this is the first demonstration of comparable activity with a glass substrate.

More advanced studies such as high‐resolution imaging and changes in protein expression have recently shown that on different topographies or within the same, some bacteria can be impaled or deformed on nanopillars, and some exhibit an enhanced oxidative stress response, resulting in mechanically ruptured and lysed cells or not.^[^ ^44^, ^46^ ^]^ This multitude of scenarios suggests that either several mechanisms exist at once for some structures, or one dominates, driven by a particularity of the topography‐bacterium pair (and likely environment). Therefore, to elucidate the underlying mechanism, we further probe the surface by SEM imaging (Figure 5d–f) and discover the significantly lower number of adhered bacteria to the nanostructures than to the controls, also observing that ≈50% of those attached cells are ruptured/lysed (see the Experimental Section). Amongst the bacteria that appear intact on the nanostructures, we note they appear flattened/deformed when resting on nanopillars, yet there are no clear signs of their disintegration (debris or cytosolic content visible). This can be attributed to cellular leakage (washed away), loss of turgor pressure, and/or stretching deformation upon adhesion (which likely is pre‐(full) rupture provided the number of dead bacteria found by fluorescent imaging which scores viability based on the membrane integrity). Analysis of the average cell surface area and its distribution (Figure 5f; see the Experimental Section) reveals an increase, further confirming the bacteria are flattened. Nonetheless, although made of glass, we cannot exclude the role of pillar flexibility due to their fine size, evidenced in Figure S23 in the Supporting Information, to contribute to the overall performance via a recently proposed energy storage–release mechanism.^[^ ^48^ ^]^ Overall, this demonstration of antibacterial glass indicates that at *p* ≈ 100 nm, *S. aureus* is killed with an efficiency matching the best reported structures in silicon, leading to bacteria lysis through stretch‐and‐rupturing and likely piercing, enhanced by the deflection of nanopillars. Further gains in performance are anticipated by adjusting the AR, however we reserve this investigation for future studies, and instead place emphasis on the potential of RSML as a fabrication tool in controllable nanoscale glass etching to achieve such functionalities.

In summary, we present a two‐step fabrication concept to realize uniform nanostructures of varying aspect ratio with high‐resolution in glass. This significantly simplifies current complex approaches while offering superior control—stemming from both the masking (pitch; mask type), and the etching, with in‐situ secondary organic mask formation (tapering/diameter/height). Overall, this constitutes a generic solution for enhancing etching selectivity to elicit deep structures templated from thin soft masks. We anticipate these qualities to drive glass fabrication in both academic and industrial settings due to attainable feature dimensions (including <100 nm; aspect ratio > 1), well‐aligned with emerging trends in the design of multifunctional surfaces. We draw attention to the method potential by balancing optical transparency, water impact‐resistance, and importantly, realize the first reported nanostructured glass surface capable of killing *S. aureus* with 81% efficiency. Despite the vast market for such antimicrobial product, scarce examples of nanostructuring exist in glass,^[^ ^49^ ^]^ making RSML particularly important to drive this field forward, by providing the tools for systematic studies via tuning nanostructures shape and aspect ratio. Overall, the ability to simultaneously attain some or all of these properties may find use in applications including solar panels, high‐rise glass buildings, food/therapeutics packaging and hospital/bathroom settings, to list a few.

## 3 Experimental Section

### Block Copolymer (BCP) Micelles Preparation

BCP micelles of PS‐*b*‐P2VP [poly(styrene‐*block*‐2vinylpyridine), Polymer Source Inc.] were pre‐assembled according to the previous report,^[^ ^27^ ^]^ with certain adaptations. To accommodate pitch ranging from ≈50 to 300 nm, four molecular weights were used: P57, P100, P200, and P400 corresponding to *M*_n_ (× 10^3^ g mol^−1^): 57‐*b*‐57, 109‐*b*‐90, 248‐*b*‐195, and 440‐*b*‐353, respectively, with the following polydispersity index (*M*_w_/*M*_n_) values: 1.05, 1.08, 1.09, 1.18 (see Section S1 in the Supporting Information for details). The polymers were mixed with anhydrous *m*‐xylene at the concentrations of 0.3–0.5% w/v by gentle stirring at 75 °C for 16 h to form spherical micelles. Subsequently, the solutions were allowed to cool to room temperature (RT), filtered (poly(tetrafluoroethylene) (PTFE) 1 µm), and stored at 4 °C.

### Nanopillars Fabrication in Glass

The fabrication scheme is presented in Figure 1a. During the first step, the pre‐assembled micelles were spin‐coated for 30 s (SCS G3 Spin Coater) onto the pre‐cleaned (acetone, isopropyl alcohol) glass wafer at RT (Fused silica JGS1, 2″‐wafer, 500 ± 25 µm; MicroChemicals GmbH). For P100, the typical spin speeds were 3 and 6k rpm, whereas for P400, we used 1–2k rpm. For masking with photoresist, please see the Supporting Information. To register the pattern in glass, reactive ion etching (RIE) was conducted using PlasmaPro NGP80 RIE, Oxford instruments, at temperature of 20 °C. First, breakthrough etch 1 was performed to tune diameter of the mask and remove PS matrix under O_2_ (20 sccm), pressure 50 mTorr, and radio frequency (RF) power 50 W. Time varied between 3 and 14 s depending on the BCP used (e.g., 3 s for P57 and 4 s for P100). Subsequently, glass was etched using CHF_3_/H_2_/Ar gases at flows 12–15, 0–6, and 45–75 sccm, respectively; under pressure of 30 mTorr and at RF power of 220 W. The optimal values were found to be 15, 5, and 65–70 sccm. Note, the Ar flow can be constant or varied to manage the deposition, diameter, and sidewall tapering. The etching depth was controlled by the etching time and could only proceed until secondary mask reached maximum diameter equal pitch. If taller structure was required, breakthrough etch 2 was performed under O_2_ plasma (conditions as above) to reduce the diameter of the secondary mask ($d_{T}^{D}$). The time of this etch was estimated based on the $d_{T}^{D}$ value and etching rate (≈2 nm s^−1^). The typical values used were 10–16 s. This completes the first etch cycle which can be iterated until a desired height is reached (Figure 1c; Figure S10, Supporting Information). Eventually, sample was cleaned under O_2_ plasma (50 sccm, RF power 200 W) for 2 min. For very high aspect ratio, an additional chamber clean between the cycles may be required due to continuous passivation of the reaction chamber.

The fabrication of double‐sided nanostructures can be found in Section S9 in the Supporting Information.

### Surface Characterization

Topological characterization of BCP patterns (diameter, pitch) was evaluated using an atomic force microscope (Dimension Icon‐PT from Bruker AXS) in tapping mode in air at room temperature. The scanning speed was 1.00 Hz s^−1^ with 256 Samples/Line. The tips were NANOSENSORS PPP‐NCHR, which have a tip radius curvature of <10 nm, tip height of 10–15 µm, and are highly doped silicon with an Al coating on the detector side. Pitch was determined by using ImageJ (https://imagej.nih.gov/ij/) software with nearest neighbor distance plugin. The scanning electron microscopy images were taken by Carl Zeiss XB1540 SEM and SmartSEM software (equipped with tilt correction) at 2–5 kV operating voltage. Prior to the imaging, the samples were sputter‐coated with Au. For both AFM and SEM imaging, at least five independent fields were measured. ImageJ was used for statistical analysis of the nanostructure dimensions such as pitch, height, diameters (with 50 quantities measured). The chemical composition was characterized by X‐ray photoelectron spectroscopy (XPS) with a Thermo Scientific K‐Alpha Photoelectron Spectrometer using monochromatic Al kα radiation at 1486.6 eV. Survey scans were collected in the binding energy range of 100–1100 eV at a pass energy of 160 eV. CasaXPS version 2.3.16 software was used for peak fitting and binding energies were adjusted to adventitious carbon (284.5 eV) for charge correction.

### Transmission/Reflection Measurements

Transmission measurements were performed by attaching the samples to the input port of an integrating sphere (Labsphere) and illuminating the samples with a collimated white light source (Labsphere, KI‐120 Koehler Illuminator). The output port of the integrating sphere was connected to a CCD spectrometer (Ocean Optics) by optical fiber. The details of the measurements are presented in Section S8 in the Supporting Information. Measurements were taken at angles of incidence between 0 and 60° in intervals of 10° by rotating the imaging sphere and light source. Reference measurements were taken at each angle to consider the angular response of the setup. Since the samples are non‐absorbing reflectance *R* was calculated as *R* = 1 − *T*. Beyond 60°, the signal‐to‐noise ratio originating from our experimental set‐up degraded significantly, hindering the measurements. We therefore use our model, (see below) and expand the analysis up to 80°. We observe significant degradation above 60° in agreement with past results,^[^ ^11^, ^13^ ^]^ resulting from the *z*‐component of the *k*‐vector going to zero for grazing angles of incidence, thereby requiring very large aspect ratio to fulfil impedance matching conditions.^[^ ^34^ ^]^

### Optical Model

The effective refractive index of the nanocones was calculated by using the Maxwell–Garnett theory as^[^ ^50^ ^]^

(3) $$n_{\text{eff}}\left(z\right)=n_{{\text{SiO}}_{2}}^{2}\frac{2\left(1-f_{\text{air}}\left(z\right)\right)n_{{\text{SiO}}_{2}}^{2}+\left(1+2f_{\text{air}}\left(z\right)\right)}{\left(2+f_{\text{air}}\left(z\right)\right)n_{{\text{SiO}}_{2}}^{2}+\left(1-f_{\text{air}}\left(z\right)\right)}$$

where *f*_air_(*z*) is the volume fraction of air in the glass/air mixture at depth *z*, given by

(4) $$f_{\text{air}}\left(z\right)=1-\frac{2\pi r{\left(z\right)}^{2}}{p^{2}\sqrt{3}}$$

To derive the previous formula, it was assumed that the nanocones are arranged in a periodic hexagonal arrangement and their cross‐section at *z* is circular and of radius *r*(*z*). The exact distribution for *r*(*z*) was extracted from the SEM images and is fitted to two linear functionals as shown in Section S9 in the Supporting Information. To model the transmission and reflection characteristics of the double‐sided structure, the generalized coherent/incoherent transfer matrix method was used.^[^ ^38^, ^39^ ^]^ Optimization for different aspect ratios covering the range 0.1 < AR < 10 is presented in Figure S15c in the Supporting Information, where it is shown that for the given pitch, antireflectance performance is optimized for any AR > 2. This design rule can then be combined for the requirements for superhydrophobicity and/or antibacterial activity, in order to create multifunctional glass. More details of the optical model and subsequent calculations can be found in Section S9 in the Supporting Information.

### Functionalization

The samples were first cleaned via sonication in acetone and isopropyl alcohol, and then subjected to an oxygen plasma (Diener Femto Plasma Etcher) in order to impart surface hydroxylation (5 min each, maximum of power generator). Immediately following this, they were immersed in a 2% v/v heptadecafluorotrimethoxysilane solution in anhydrous toluene at room temperature for 24 h, washed, and subsequently annealed at 120 °C for 30 min.

### Wetting Characteristics

Both advancing and receding contact angles were measured using a custom designed goniometry setup. The setup consists of syringe pump (Cole–Parmer Single‐syringe infusion pump), a needle (BD PrecisionGlide needles, 21G), and an imaging device (Thorlab, model DCC1240). Droplets of ≈30 µL were deposited onto the surfaces and further extracted using the syringe pump to measure advancing and receding contact angle, respectively. The videos taken during droplet deposition and extraction were processed through a Matlab script for contact angle measurements,^[^ ^51^ ^]^ which is available from the corresponding author upon reasonable request.

Droplet bouncing was characterized by releasing a droplet of radius *r* = 0.9 mm (≈3 µL) from a pipette onto the sample from a height of 1 cm, giving rise to an initial velocity *V* = 0.33 m s^−1^ and *W* *e* = 1.33 (where *We* is the dimensionless Weber number, which is the ratio between deforming inertial forces and stabilizing cohesive forces of a fluid). A high‐speed camera (Phantom V411 fitted with a macro lens) was used to record and count the number of bounces. Freeze frames were taken, and the height of the droplet over time was measured using ImageJ.

Droplet impact experiments were carried out by dispensing droplets with an unperturbed radius *r*_0_= 1.35 mm from a needle (BD PrecisionGlide, 21G) mounted at different heights to obtain a range of impact velocities, and recorded using a high‐speed camera. Freeze frames were taken, and the diameter of the droplet as a function of time was measured using ImageJ.

### Bacterial Cultures

A model Gram‐positive bacterium, *S. aureus* ATCC 6538 was used in this study as it is recommended by the ISO standard (International Organization for Standardization) JIS Z 2801 (2010) for assessing the antibacterial properties of materials. Bacteria were maintained frozen at −70 °C and cultured on brain heart infusion (BHI) agar. To prepare the inoculum for application to the materials, bacteria were cultured to mid‐exponential phase in BHI broth, aerobically, at 37 °C and with shaking at 250 rpm. The bacteria were recovered by centrifugation, washed twice in phosphate‐buffered saline (PBS), and diluted to 10^7^ colony forming units (cfu) mL^−1^, unless stated otherwise.

### Determination of Bacterial Viability

The antibacterial properties of the nanostructured surfaces were quantitatively evaluated using an adhesion based‐assay. First, test surfaces and controls (flat fused silica; both 1 cm^2^) were flame‐sterilized and an inoculum volume of 25 µL (2.5 × 10^5^ cfu) applied by dropping onto, followed by incubation for 18 h at room temperature (25 °C) in a high humidity achieved by placing soaked filter in the petri dish. Subsequently, the non‐attached bacteria were washed off and collected, serially diluted to obtain 30–300 colonies per plate, spread on agar plates, and incubated at 37 °C for 18 h. Finally, the colonies were counted, and bactericidal efficiency (BE) determined according to the following equation

(5) $$BE=100-\left(\frac{V_{x}}{V_{ctrl}}\right)\;\cdot \;100$$

where *V*, x, and ctrl refer to the number of viable cells, experimental and control sample, respectively. The experiment was performed three times with 3–6 technical replicates.

The viability of the bacteria that remained attached onto the surfaces was evaluated by confocal laser scanning microscopy (CLSM) using LIVE/DEAD staining. The surfaces were rinsed with 1x Tris‐buffered saline (TBS) to remove traces of the growth medium (known to quench the fluorophores), followed by staining with the LIVE/DEAD BacLight kit (L7012, Invitrogen) per the instructions. The kit contains SYTO 9 (green) and propidium iodide (PI, red) dyes that stain the cells depending on their membrane integrity. Bacterial cells with intact membranes are stained green, whereas cells with a damaged membrane (that are considered to be damaged or dead) are stained red. Image acquisition was performed by means of a CLSM (BioRad Radiance2100, Zeiss, Welwyn Garden City, Herts, UK) with 60x objective, and Bio‐Rad image analysis software. The cells were visualized by using 488 and 543 nm excitations and HQ515/30 and E600LP emission filters. Two color channels, green and red, were acquired for each image. To remove the fluorescent background noise from the image, brightness levels in every channel were adjusted. The images were processed by ImageJ for BioRad CLSM imaging by counting cells from a minimum of five fields of view across each surface. The experiment was performed three times with two replicates. The bactericidal efficiency was expressed as a percentage of non‐viable cells out of the total counted cells.

### SEM Imaging of Cell–Material Interactions

Bacterial suspensions containing 10^9^ cfu mL^−1^ (to generate statistically reliable data) were interacted with the samples for 18 h. Next, the samples were washed twice with 1 × PBS, and subsequently fixed using 3% glutaraldehyde solution in 0.1M sodium cacodylate buffer (4 °C, 16 h). After fixation, samples were dehydrated in an ethanol series of 20%, 50%, 70%, 90%, and 100% (v/v) for 10 min each. Subsequently, the samples were immersed in hexamethyldisilazane^[^ ^52^ ^]^ (HMDS, Sigma Aldrich) for 5 min, air‐dried, sputter‐coated with Au, and imaged using SEM (Carl Zeiss XB1540) at 2 kV. The images acquired were further analyzed in terms of (i) a number of cells adhered onto to the surface, (ii) the proportion of cells ruptured (when a clearly compromised membrane was observed and/or cellular content was present external to the cell), and (iii) an average cell surface area, *A*—to express quantitatively if there is a difference in bacterial morphologies between controls and tested samples. Here, clearly disintegrated or dividing bacteria with visible septum were dismissed. ImageJ was used to analyze the images and to accurately determine the surface area of each bacterium, the cells were treated as ellipses. Therefore, the semi‐minor and semi‐major axes (*a* and *b*, respectively) of 50 cells on both flat and nanostructured surfaces were measured, and the area was calculated according to Equation (6):

(6) A=πab

## Conflict of Interest

The authors declare no conflict of interest.

## Supporting information

Supporting Information

Supplemental Video 1

Supplemental Video 2

Supplemental Video 3

Supplemental Video 4

Supplemental Video 5

## Data Availability

The data that support the findings of this study are available from the corresponding author upon reasonable request.
