# Supplementary material for: Bioinspired Multifunctional Glass Surfaces through Regenerative Secondary Mask Lithography
Source: Adv Mater. 2021 Sep 13;33(43):2102175. doi: 10.1002/adma.202102175 (PMC11469219; doi:10.1002/adma.202102175)
Supplement: Supplementary file 1 — Supporting Information [file ADMA-33-2102175-s004.pdf]

© 2021 Wiley-VCH GmbH

ADVANCED MATERIALS

Supporting Information

for *Adv. Mater.,* DOI: 10.1002/adma.202102175

Bioinspired Multifunctional Glass Surfaces through Regenerative Secondary Mask Lithography

*Martyna Michalska, Sophia K. Laney, Tao Li, Mark Portnoi, Nicola Mordan, Elaine Allan, Manish K. Tiwari, Ivan P. Parkin, and Ioannis Papakonstantinou**

Supporting Information

Bioinspired Multifunctional Glass Surfaces Through Regenerative Secondary Mask Lithography

*Martyna Michalska†, Sophia Laney†, Tao Li†, Mark Portnoi, Nicola Mordan, Elaine Allan, Manish K. Tiwari, Ivan P. Parkin and Ioannis Papakonstantinou**

†These authors contributed equally to this work

*i.papakonstantinou@ucl.ac.uk

This supporting information contains additional data and follows the development of the accompanying paper.

Table of Content:

	Title	Page
Supporting Methods		*3*
	Fabrication of glass nanostructures using photoresist mask	*3*
Section S1	*Block copolymer patterns*	*4*
Figure S1	*Characterization of block copolymer patterns*	*6*
Figure S2	*Block copolymer mask after Breakthrough Etch 1 (Step 2.1)*	*6*
Section S2	*Glass etching chemistry*	*7*
Figure S3	*A schematic of glass etching chemistry*	*8*
Section S3	*Pseudo-Bosch process*	*9*
Figure S4	*Exemplary structures obtained by Pseudo-Bosch process*	*9*
Section S4	*Secondary mask evolution and structure tapering*	*10*

	with $R^D \gg R^S$	
Figure S5	*Secondary mask evolution and structure tapering when $R^D \gg R^S$.*	*11*
Figure S6	*The glass etching rates when varying argon flow*	*11*
Section S5	*XPS analysis*	*12*
Figure S7	*X-ray photoelectron spectroscopy – survey spectra*	*12*
Section S6	*Method validation – various BCPs*	*12*
Figure S8	*Exemplary glass nanopillars*	*13*
Figure S9	*Glass nanopillars templated from BCP P400*	*14*
Figure S10	*Fabrication process of glass nanopillars templated from BCP P400*	*14*
Figure S11	*Control experiment*	*15*
Section S7	*Method versatility*	*15*
Figure S12	*RSML applicability*	*16*
Figure S13	*The effect of an RSML-mediated etch on nanostructure diameter, using a photoresist mask*	*17*
Section S8	*Mechanical stability*	*18*
Figure S14	*Tape-peel test*	*19*
Section S9	*Optics*	*19*
Figure S15	*Optical Model*	*20*
Section S10	*Double-sided patterning*	*21*
Figure S16	*Double-sided glass processing*	*22*
Table S1	*Critical nanocone parameters used in the optical model*	*23*
Figure S17	*Haze measurements*	*24*
Section S11	*Wettability*	*24*
Figure S18	*The effect of sharpening on the sample wettability*	*25*
Figure S19	*Rebound characterization of a water droplet*	*26*
Figure S20	*Capillary pressure versus penetration percentage*	*28*
Section S12	*Model of interactions between nanopillars and bacteria cells*	*28*

Figure S21	*Schematic of S. aureus adhered to the surface*	*29*
Figure S22	*Effect of geometry of nanopillars on the interactions with S. aureus*	*31*
Figure S23	*S. aureus cells attached onto glass nanopillars*	*32*
References		*33*

Supporting Methods

Fabrication of Glass Nanostructures Using Photoresist Mask. 1) Generation of the Photoresist Pattern: The photoresist pattern was generated by imprinting a silicon master ‘stamp’ [Master 1: p = 350 nm, h = 400 nm, and Master 2: p = 110 nm, h = 95 nm] onto a fused silica substrate via nanoimprint lithography (NIL). Prior to imprinting, the master stamp was functionalized with a polydimethylsiloxane (PDMS) brush layer according to a previously reported method,[1] to facilitate demolding. Briefly, oxygen plasma was applied to activate the surface which was subsequently grafted with short chain PDMS using 1:10:0.27 v/v/v ratio of dimethyldimethoxysilane : isopropanol : H_2SO_4 (>95%) mixture. The substrate was placed on a hot plate (75°C) and the solution was drop-casted atop for 15 s, followed by washing with deionized water, isopropanol, and toluene.

To generate a positive-tone replica of the pattern, a two-step nanoimprint was conducted. The NIL process was performed on EITRE® 3 (Obducat). The intermediate polymer (IPS, Obducat) stamping process was operated at 145°C and 40 bars for 20 s (Master 1) and 150°C and 20 bars for 20 s (Master 2). Transfer of the negative pattern into the photoresist (STU-7, Obducat) was operated at 70°C and 30 bars for 240 s with 60 s of UV curing. The photoresist dilution and spin coating speed were adjusted per each master so that there was a minimum of residual layer (<20 nm).

2) Transferring the Photoresist Pattern into SiO_2: To register the pattern in glass, reactive ion etching (RIE) was conducted as described in Methods section with certain modifications. Master 1 – briefly, after removal of the residual photoresist layer under O_2 plasma for 40 s (breakthrough etch 1; time dependent on residual layer thickness), the glass etching was performed in the absence of H_2 for 14 min, yielding nanopillars with the depleted mask (tens

nm). Subsequently, H_2 (6 sccm) was introduced into the gas feed to induce self-masking and allow the structures to be etched further. The substantial H_2 amount was introduced in order to emphasize the deposition effect. Master 2 – Breakthrough etch 1 was conducted for 17 s, after which etching was alternated in respect to Ar flow within 65-75 sccm for a total time of 25 min.

Supporting Sections

Section S1. Block Copolymer Patterns

Block copolymers (BCP) can self-assemble to form regular patterns at dimensions as small as sub-10 nm, which are unattainable through conventional lithographic techniques.[2] However, to elicit certain morphologies (spherical, cylinders, lamellae etc.) and maintain quality of the resulting pattern (homogeneity), the BCP phase diagrams need to be carefully followed by strictly controlling the self-assembly conditions. In a typical BCP lithography process, a co-polymer brush layer is first deposited,[3] in order to ensure the perpendicular alignment of BCP domains to the substrate during the subsequent thermal or solvent annealing.[4] Additionally, other steps may be required such as development.[5] Unlike those processes, we take a significantly simpler approach by pre-assembling PS-*b*-P2VP (as an example) inverse micelles, which when spin-coated, form patterns within seconds at room temperature,[6,7] and therefore does not require the aforementioned pre-processing or post-processing steps. It is noteworthy that the annealing step indeed increases the cost of fabrication and its complexity, and some of the aspects might be challenging to implement in industry.

We explore four polymers (P) of M_w: P57, P100, P200, and P400 (M_w / kg mol^{-1} = 57-*b*-57, 109-*b*-90, 248-*b*-195, and 440-*b*-353, respectively), by dissolving the polymers in xylene isomers, and we find the most suitable dimensions of micelles and highly reproducible results for anhydrous *m*-xylene. Note, *p* and P refer to pitch and type of polymer, respectively. The pattern characterization by atomic force microscopy (AFM) revealed hexagonally-packed micelles of excellent uniformity, as shown in Figure S1a. The choice of solvent is extremely important. Here, we show the same P100 polymer dissolved in regular *m*-xylene, which results in micelles of a bimodal distribution (Figure S1b). Importantly, bimodality can enable binary or hierarchical structures, for instance, whereby two different diameters or heights of the feature can be achieved. Alternatively, a controlled disorder in the layout may be introduced by complete elimination of the second geometrical population during the subsequent etching. The height of

the micelles is influenced by the humidity during the spin-coating process through swelling of the P2VP core with water, however as discussed later, the nature of our etching process ensures this is not problematic [BCP mask is immediately embedded within a secondary mask during our *regenerative secondary mask lithography (RSML)* process, thus selectivity is maintained].
By varying spin speeds, a different pitch can be achieved as presented in Figure S1c for P57, P100, and P400. The chart nicely summarizes the achievable pitch range being ~50-300 nm. Note, for very high M_w, there is no-linear dependence in respect to spin speeds as well as the pattern quality suffers when too rapid spin-casting is applied.

Oxygen plasma is used as breakthrough etch 1 in order to remove the PS matrix and uncover the underlaying material to be etched (Step 2.1 in Figure 1a; Figure S2). Whilst a combination of M_w, polymer concentration and spin speed control the pitch, Step 2.1 tunes the diameter of the micelles (dependent on M_w and solvent used), by reducing it when increasing the etching time. However, longer the time, shorter the micellar bumps become which in turn, generally affects the etching selectivity. Nonetheless, since in our process the mask is being embedded within the secondary-protective mask, this issue can be alleviated. Furthermore, the mask diameter can be even enlarged – a unique opportunity unlocked through our process – when a high hydrogen content is introduced to the gas feed in the subsequent glass etching. In order to retain the critical dimensions, though, after a given diameter is reached, the conditions for anisotropic etching should be immediately introduced – typically, through lowering H_2 or increasing Ar.

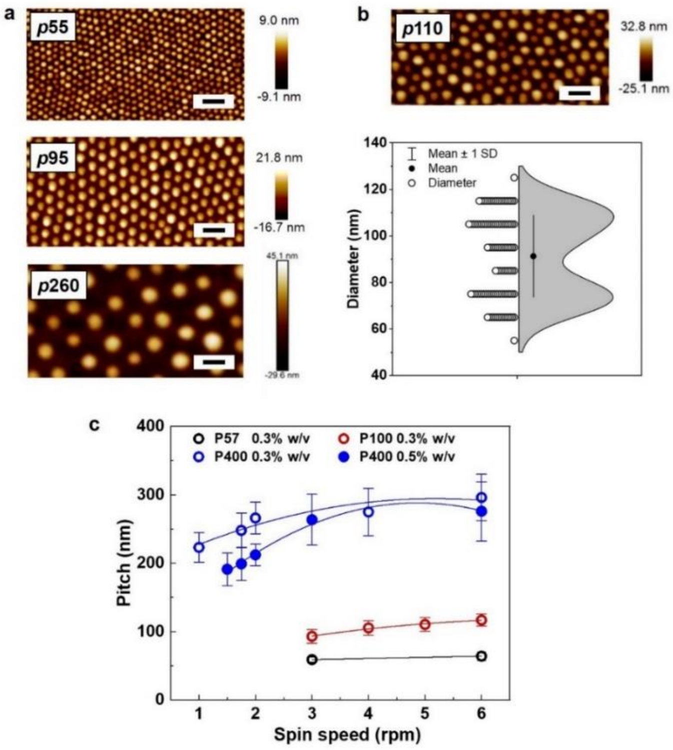

Figure S1 Characterization of BCP patterns. a, AFM images presenting hexagonally-packed PS-*b*-P2VP micelles on glass substrates with three pitches *p*: 55, 95, and 260 nm. The resulting patterns of high quality were obtained by using 3% (w/v) solutions in anhydrous *m*-xylene with polymers of various molecular weight M_w: P57, P100, and P400, respectively; and their spin-coating at 3k, 6k, and 2k rpm, respectively. Scale bars are 200 nm. The color scales correspond to the height of the micelles. **b,** AFM image of P100 solution in regular *m*-xylene spin-coated at 6k rpm showing the pattern characterized by bimodal distribution, clearly indicated by the graph below (open circles refer to data). This example highlights the importance of solvent choice/quality. Additionally, it presents a pathway for bimodal structuring. **c,** Pitch as a function of spin speed for P57, P00, and P400 at the concentration of 3% w/v. Additionally, 2% w/v concentration of P400 is plotted showing how the range of the attainable dimensions can be further tuned. Note, with higher $M_{w,}$ there is no-linear dependence in respect to spin speeds.

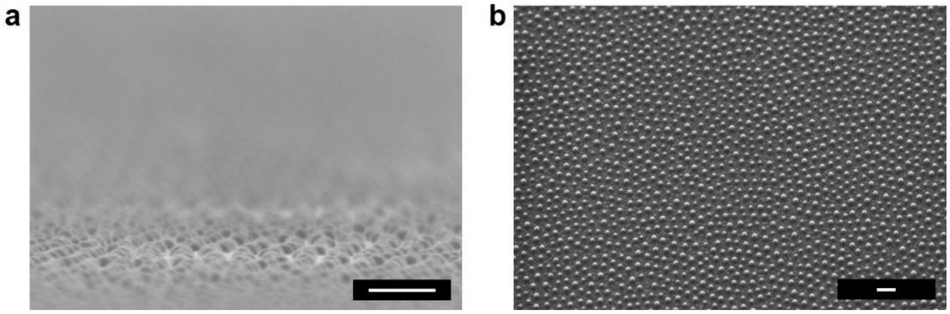

Figure S2 BCP mask after breakthrough etch 1 (Step 2.1). SEM images of BCP P100 micelles spin-coated at 6k rpm and after 4 s of breakthrough etch 1 to remove PS matrix and

uncover the underlying substrate to be etched. **a,** 2° tilted view. **b,** Top view. Note, hexagonal layout. Scale bars 200 nm.

Section S2. Glass etching chemistry

When using CHF_3 etching gas during SiO_2 plasma etching, the free fluorine radicals (F*) and other radicals such as CF_x* are generated according to the following reactions:

$$e^- + CHF_3 \rightarrow CHF_2^+ + F^* \text{~~+ radicals~~} + 2e^- \quad (1)$$

$$e^- + CHF_3 \rightarrow CF_2 + HF + e^- \quad (2)$$

$$SiO_2 + xF^* \rightarrow SiF_x + O_2 \quad (3)$$

$$3CF_x^* + 2O_2 \rightarrow CO + CO_2 + COF_2 \quad (4)$$

$$3CF_x^* + 2SiO_2 \rightarrow 2SiF_x + CO + CO_2 + COF_2 \quad (5)$$

F* is the reactive fluorine atom (radical) and SiO_2 is glass.

During plasma-decomposition of the CHF_3 etchant, reactive fluorine atom F*, HF, and other radicals and ions are generated (reactions 1-2; Figure S3). The energetic heavy Ar ions cause the Si-O bonds to break, providing the disassociated silicon that can now react with fluorine radicals resulting in SiF_x formation (SiF_4 is volatile, hence it is evacuated from the chamber). Of note, the ion bombardment can also promote the desorption of some species chemisorbed on the surfaces, thus affecting the etch rate, degree of polymerization, as well as anisotropy. It can be seen in reaction 3 that the silica etching consumes the F atom but frees the oxygen. The resultant fluorocarbon CF_x radicals possess high sticking coefficients and typically deposit a polymer film on all surfaces, thus acting like an inhibiting layer for the further etching but also like promoters of anisotropic etching when depositing on the sidewalls. However, the presence of oxygen leads to the reactions with the polymer and formation of volatile CO, CO_2 and COF_2 so they can be also evacuated from the chamber (3). It also leads to a release of some of the F atoms which can aid silica etching (4) and they generally enrich the F content hence, increasing the F/C ratio.[8] The F/C ratio is important to monitor with a general trend that the lower the ratio, the higher the concentration of CF_x species at the expense of F, which switches plasma into more polymerizing.

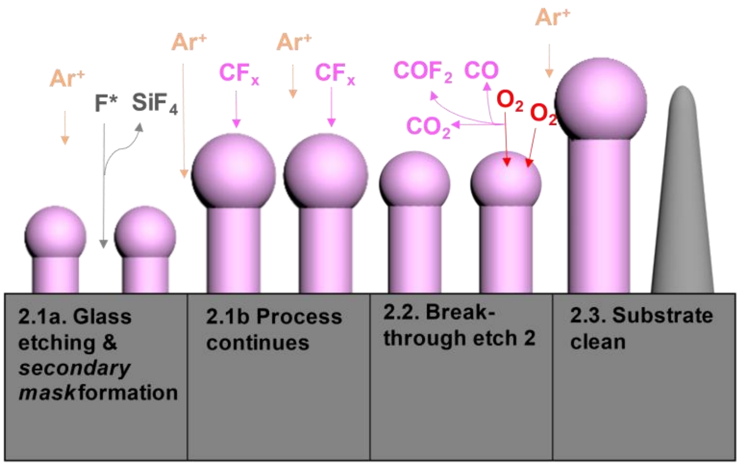

Figure S3 A schematic of glass etching chemistry. Generation of the SiO_2 nanopillar through RSML is depicted. Starting from the BCP mask (not shown), the addition of H_2 into the etching chemistry (CHF_3/Ar) increases the generation of HF, thus lowering the F/C ratio; resulting in a more polymerizing plasma with increased CF_x deposition (2.1a-b). As the secondary mask builds up around the BCP to form mushroom-like structures, a brief oxygen plasma is applied which refines the diameter (2.2). This enables further etching to proceed, with self-regeneration of the mask to yield tall SiO_2 nanopillars. A final oxygen plasma removes the deposition to form high-aspect ratio SiO_2 nanopillars (2.3).

On the other hand, the presence of hydrogen in the feed-in monomer and its further addition to the gas feed, remarkably decreases the amount of fluorine which is scavenged by these hydrogen atoms to form HF. This results again in an increase of CF_x species with a possibility to switch plasma from etching to polymerizing.[9] However, HF reacts with silica (5) which aids the etching:

$$SiO_2 + 4HF \rightarrow SiF_4 + 2H_2O \qquad (6)$$

In anisotropic etching, vertical sidewalls are the region where reactions are less frequent with also less exposure to ion bombardment, hence polymerization builds up on these locations. Such a process is called sidewall passivation in which the lateral etchings are prevented and steep sidewall profiles are realized; the Bosch process of silicon etching is a prominent example. In our case, additionally, a polymer buildup takes place on top of the structures yielding mushroom-like pillars. This shape originates from the BCP mask becoming embedded within the polymer, which we utilize here as a secondary mask in our RSML process. This mechanism prevents the BCP consumption and enables the etching selectivity.

Section S3. Pseudo-Bosch process

By altering H_2 content introduced to the gas feed (CHF_3:Ar 12 sccm: 38 sccm), broadly, we switch the plasma from etching to more polymerizing or *vice versa* (H_2 increase or decrease, respectively). When starting with higher H_2 ($CHF_3:H_2$ = 3.0, for instance), we induce self-masking originating from an excessive deposition of carbopolymer layer. By gradual hydrogen decrease (to $CHF_3:H_2$ = 3.3), we slowly reverse the plasma to become more etching so that the process behaves like a pseudo-Bosch silicon etching,[10] where the silicon anisotropic etching is alternated with the polymer passivation, which protects the sidewalls from the etching. Through such an approach, we successfully achieved nanopillars of h = 840 nm at p = 200 nm, as shown in Figure S4. However, at smaller pitch, this method was more challenging to control which prompted us to turn our attention onto process control by varying Ar content.

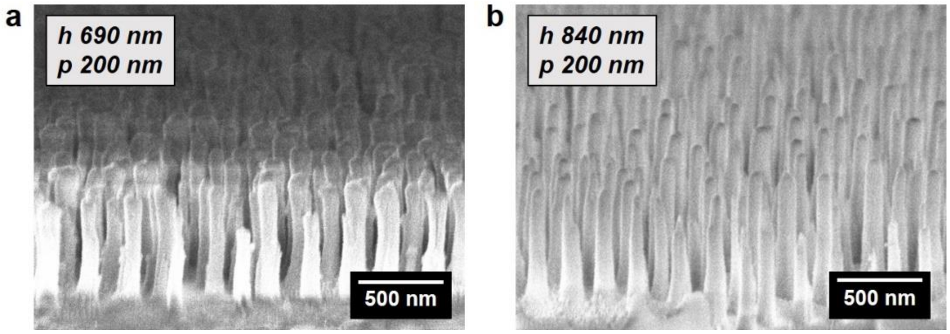

Figure S4 Exemplary structures obtained by Pseudo-Bosch process. a,b, SEM images presenting glass nanopillars obtained by our pseudo-Bosch process. The structures were templated from BCP P200 (M_w / kg mol^{-1} = 248-*b*-185) with the resulting p = 200 nm. After the breakthrough etch 1 for 7 s, the glass etching was performed in three stages for 10, 10, and 5 min by decreasing H_2 from 4, 3.8, to 3.6 sccm respectively, under constant CHF_3:Ar = 12:38 sccm. Additionally, a step of oxygen clean followed by SF_6 etch for 1 min was performed in (**b**).

It is noteworthy that pseudo-Bosch approaches were also attempted in glass etching in order to address a problem of feature distortions during high-aspect ratio nanopattern generation (*e.g.*, twisting, tilting, surface roughening).[11] For example, advanced cyclic etching was proposed, where deposition and etching cycles were alternated, resulting in a successful etch of high-aspect ratio nanoholes from an amorphous carbon layer of 550 nm.[12] In another approach, the contact hole distortions caused by uncontrolled carbopolymer deposition at the oxide-mask interface were improved through introduction of an *in-situ* polymer removal step by oxygen.[13] However as mentioned above, controlling etching of nanopillars/cones at smaller pitch (<100 nm),

templated from easily-degradable thin soft masks (~25 nm), poses its own set of challenges; which we successfully address by a non-switching deposition/etching process.

Section S4. Secondary mask evolution and structure tapering with $\boldsymbol{R^D} \gg \boldsymbol{R^S}$

Secondary mask evolution and etching profile are dependent on the deposition rate R^D, and to allow for anisotropic/directional etching at the nanoscale, this value needs to be optimal; otherwise, the maximum height that structures can reach becomes quickly limited by the etch stop when: (i) d_T^D reaches maximum value, equivalent of pitch, or (ii) excessive tapering leads to bridging bases of the neighbouring pillar. The evolution of such an effect when $R^D \gg R^S$**,** and its consequences are depicted in Figure S5. After 4 min of etching, d_T^D approaches its maximum so the resulting pillars are still nicely separated. However, as the etching continues (8 min), an uneven structuring appears, manifested by the presence of pillar doublets connected by the common base (red arrow) and a non-linear baseline (dashed line). The determined R^D that led to the premature etch-stop is 10 nm/min.

Since we balance the processes by adjusting the R^S, it is important to elucidate its impact on the etching rate. Figure S6 presents data for the typical range of the values of Ar flow in use, and we can convincingly state that the effect of Ar flow is negligible across the studied conditions.

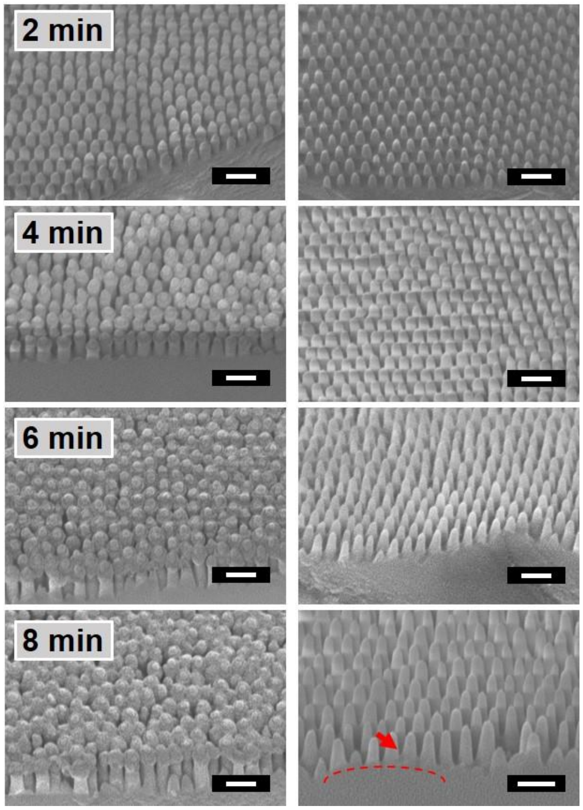

Figure S5 Secondary mask evolution and structure tapering when $\boldsymbol{R^D \gg R^S}$. SEM images 45°-tilted of the evolving glass nanopillars with deposition (secondary mask; left panel) and after O_2 clean (right panel). The structures were templated from BCP P100 with the resulting p = 95 nm. Due to the high R^D=8±1 nm/min, the secondary mask has reached its maximum diameter after 6 min. Additionally, both low etching directionality, which led to an increased tapering, and maximum $\boldsymbol{d_T^D}$ reached did not permit further etching to result in well-resolved array (8 min). Note uneven baseline (red dashed line) and red arrow indicating unseparated neighbouring pillars. Scale bar 200 nm.

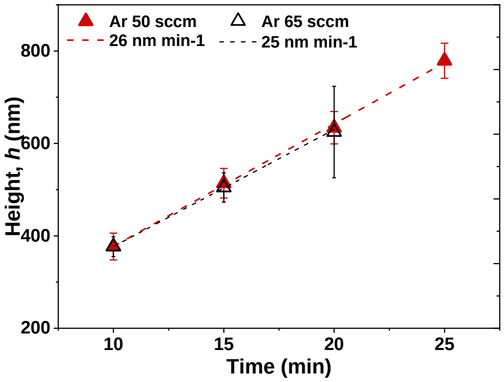

Figure S6 The glass etching rates when varying argon flow. Nanopillars templated from BCP P400 were etched using two gas mixtures with variable content of Ar (CHF_3:H_2:Ar=3:1:10 and CHF_3:H_2:Ar=3:1:12; Ar 50 and 65 sccm, respectively). The etching rates were found independent from argon flow within the tested range.

Section S5. XPS analysis

To track the chemical composition of the organic mask as a function of oxygen breakthrough time, XPS analysis was performed. Figure S7 presents representative XPS survey spectra, which served as a data source for plotting the surface composition of the samples investigated in Fig. 3c. Each sample of nanostructured glass with the secondary mask (carbopolymer deposition) was exposed to varying times of oxygen plasma (breakthrough etch 2): 0, 8, 10, 12, 16, and 30 s. Three different areas of each sample were surveyed by XPS and elementary composition was determined using Casa XPS software.

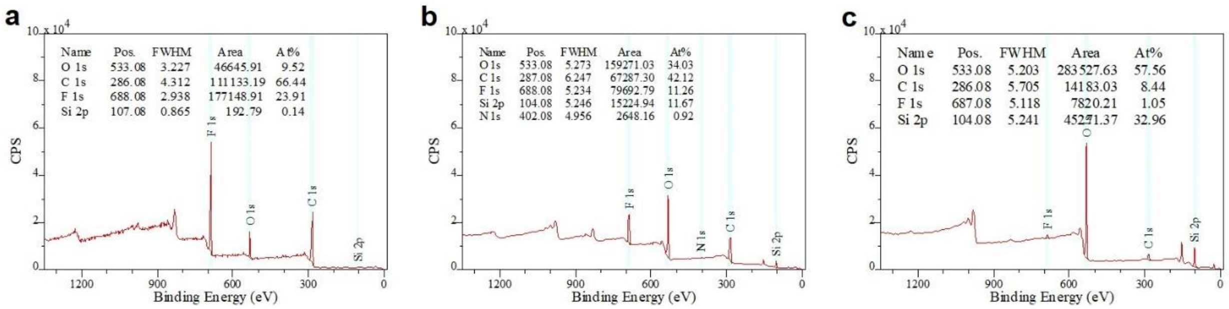

Figure S7 X-ray photoelectron spectroscopy – survey spectra. XPS survey spectra of nanostructured glass with carbopolymer deposition layer at the end of the etching during cycle n=1 (**a**), after 10 s of breakthrough etch 2 (**b**) and without deposition, after 30 s of breakthrough etch 2 (**c**).

Section S6. Method validation – various BCPs

Following the fabrication demonstrated in Figure 1, we show here the excellent uniformity of the produced arrays with p =110 nm after 1 and 3 cycles (Figures S8a and 8b, respectively). A further advantage of our process is demonstrated in Figure S8c, where we apply the same optimization to micelles with p = 95 nm – obtained through increasing the concentration of P100 in solution, whilst maintaining the same spin speed. The achieved structures of sub-100 nm pitch have an AR = 6.

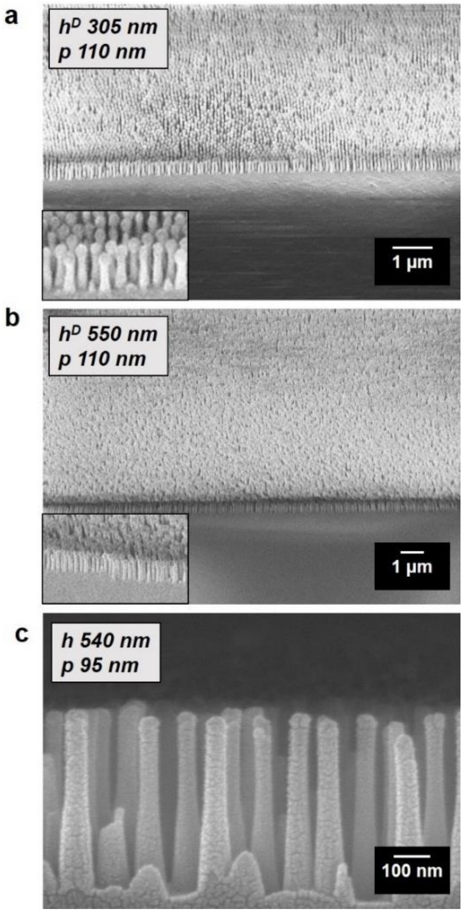

Figure S8 Exemplary glass nanopillars. a,b, Glass nanopillars with *p*=110 nm at the end of cycle n = 1 (**a**) and n = 3 (**b**) showing the uniformity across the surface. **c,** Glass nanopillars with *p* = 95 nm and *h* = 540 nm obtained through our process. Note the even heights of the presented structures.

Applying now the concept to a mask of larger pitch (P400; *p*=260 nm), three different Ar flows were tested; 50, 55, and 65 sccm. Assessing the structures after 20 min of etching at these conditions, revealed that for Ar = 65 sccm, the process had reached a maximum, resulting in the complete removal of mask, and destruction of the glass pillars (SEM micrographs not shown). Proceeding instead with Ar = 50 sccm, which provides an R^D slightly greater than R^S, the structures were assessed after 10, 15, and 25 min, as shown in SEM images in Figure S9a-c; where the quality and uniformity of the obtained arrays is evident. Additionally, the evolution of the height and top diameter with and without deposition over time is shown in Figure S10. In order to etch further, we performed an oxygen breakthrough of 18 s, which was calculated from the known etch rate, and the d_T^D. For the subsequent etch, the Ar flow was increased to 55 sccm for a further 8 min in order to induce greater anisotropy and obtain structures with a height >1

μm, possessing more vertical sides near the base, and tapered sides leading to the apex (Figure S9d).

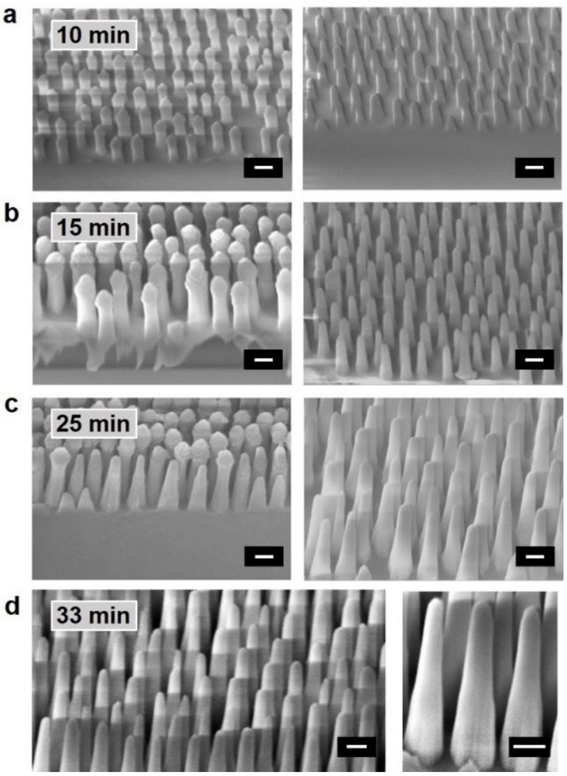

Figure S9 Glass nanopillars templated from BCP P400. a-c, SEM tilted images of evolving glass nanopillars with carbopolymer deposition (left panel) and cleaned by O_2 plasma (right panel). The process was optimized for BCP P400 and resulting pitch *p*=260 nm. Nanopillars after 10 (**a**), 15 (**b**), and 25 min (**c**) of the first etching cycle were obtained by using CHF_3:H_2:Ar = 3:1:10. **d,** SEM tilted image of glass nanopillars. After 25 min, the breakthrough etch (18 s) was applied, and the second etching cycle for 8 min was followed using CHF_3:H_2:Ar = 3:1:11. The resulting structures possess *h* = 1080 μm. Scale bars 200 nm.

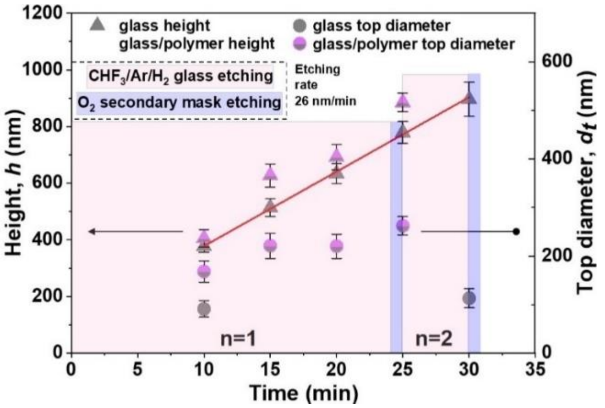

Figure S10 Fabrication process of glass nanopillars templated from BCP P400. Example of nanostructure evolution during *n*=2 cycles, monitored by changes in height and top diameter,

with and without deposited carbopolymer. The linear fit (red line) indicates etching rate of 26 nm/min.

The importance of the breakthrough etch 2 (reduction of secondary mask) is highlighted in Figure S11. There, glass templated from P400 was etched for 25 min under Ar=50 sccm (end of the first cycle), and next, 18 s of oxygen plasma was applied *or not* before proceeding with additional 7 min of glass etching (Figure S11a,b and 11c,d, respectively). As can be seen, the secondary mask is smaller at the end of total etching in Figure S11a than that in 11c, and the obtained structures are taller, well-separated, and with an even baseline (Figure S11b). The structures in Figure S11d, on the other hand, are shorter and exhibit an uneven baseline.

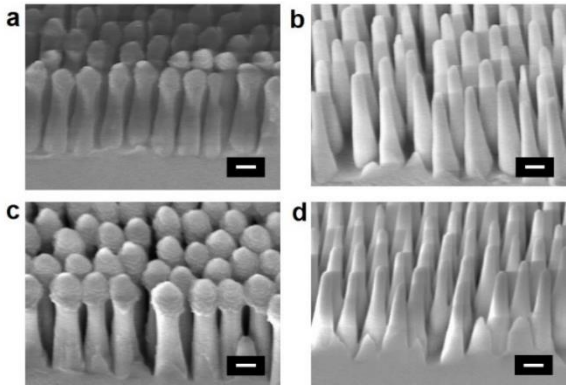

Figure S11 Control experiment. a,**b** SEM images of the pillars with deposition and cleaned, respectively, which were templated from the BCP P400. At the end of the first cycle (n=1), the sample was subjected to the breakthrough etch 2 (18 s) to refine the secondary mask, and thus allow the subsequent etching cycle (n=2) to proceed. **c,d** SEM images of the pillars with deposition and cleaned, respectively, which were processed following the same recipe as (**a,b**) but without the breakthrough etch 2. Note the difference in deposition and uneven morphology of the resulting structures, highlighting necessity of the breakthrough etch 2. Additionally, pillars in (**d**) are shorter than those in (**b**), suggesting that etching stopped during this process. Scale bars 200 nm.

Section S7. Method versatility

We now demonstrated how to successfully attain glass nanostructures from a BCP mask with sub-100 nm features and limited height through implementation of all the discussed process controls and RSML cycles (Figure S12a). However, the principles of the method and its dynamic tunability can be leveraged in other pre-pattern scenarios (in respect to pitch and height), which we show on the example of photoresist. We use nanoimprint lithography (NIL) as an alternative approach to generate scalable etch-mask with sub-100 nm features, while allowing to mitigate

BCP's shortcomings through a possibility to yield taller photoresist mask (Figure S12b; *h*~100 nm). Due to the greater initial height of the soft mask in comparison to BCP (here four times), less protection/deposition is required, and hence more sputtering is allowed during one cycle. Therefore, sufficient control of the secondary mask diameter can be attained through variation of the Ar flow, without necessitating an oxygen breakthrough to reduce it. However, if structures of greater diameter are sought, then multiple cycles would be required. Important to note however, is that although NIL permits soft masks of higher aspect ratio, above a certain threshold, an attractive force between pillars adjacent to each other induce tip-to-tip lateral collapse (Figure S12c); determined by the distance between the nanopillar masks, their surface energy, Young's modulus, and Poisson's ratio.[14] Consequently, the subsequent etching yields highly uneven structures (Figure S12c). Thus, the issue of soft mask degradation cannot be mitigated by merely increasing its height; again, confirming the need for RSML in this context.

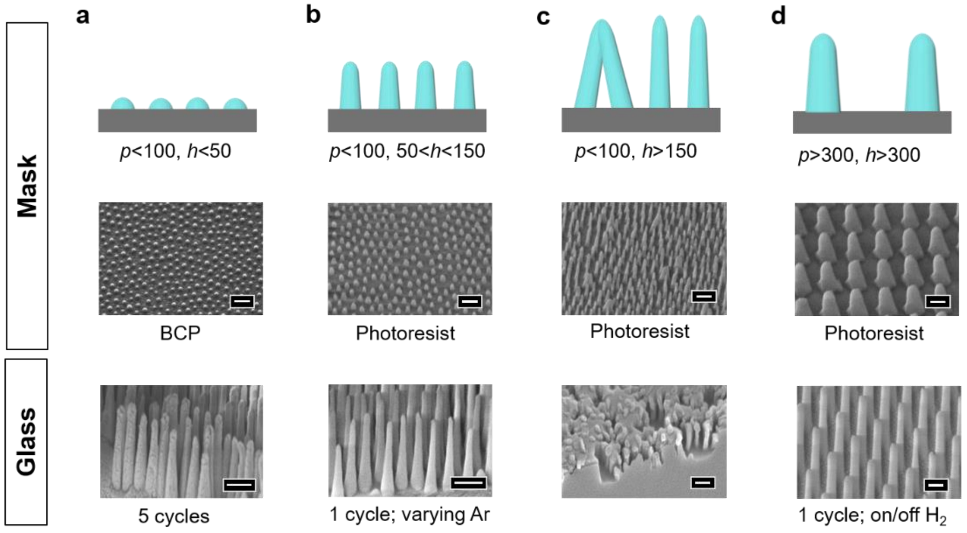

Figure S12 RSML applicability. Schematics and 45°-tilted SEM images of soft masks of varying pitch/height (approximate ranges) and the resulting glass nanostructures obtained via RSML. Depending on the pre-pattern dimensions, the process was adapted accordingly. **a,** For BCP soft masks of *ca.* p<100 nm and h<50 nm, multiple cycles of RSML are required in order to attain high-aspect ratios as discussed in the main text. **b,** For photoresist masks with *ca.* p<100 nm but height within the range 50-150 nm, only one cycle of RSML with varying Ar flow is required to attain high aspect-ratios. **c,** Increasing the height of the photoresist mask to >150 nm leads to pillar-mask leaning, and upon etching gives rise to uneven structuring. **d,** For soft masks with greater pitch (*ca.* p>300 nm) and height (*ca.* h>300 nm), etching can proceed in one cycle by varying deposition (on/off). Scale bars = 200 nm.

Finally, we demonstrate the case for a photoresist mask with both a greater pitch and height (Figure S12d; p=350 nm and h~380 nm). We etch initially in the absence of H_2 (Figure S13a) until there is significant mask consumption (Figure S13a(ii)), meaning any further etching under such conditions would result in pillar destruction. At this point, we introduce the RSML concept to induce a secondary mask (Figure S13a(iv)), and attain structures of higher aspect ratio. This also highlights that RSML can be implemented not only at the beginning of the process, but also part-way through in order to re-build a depleted mask.

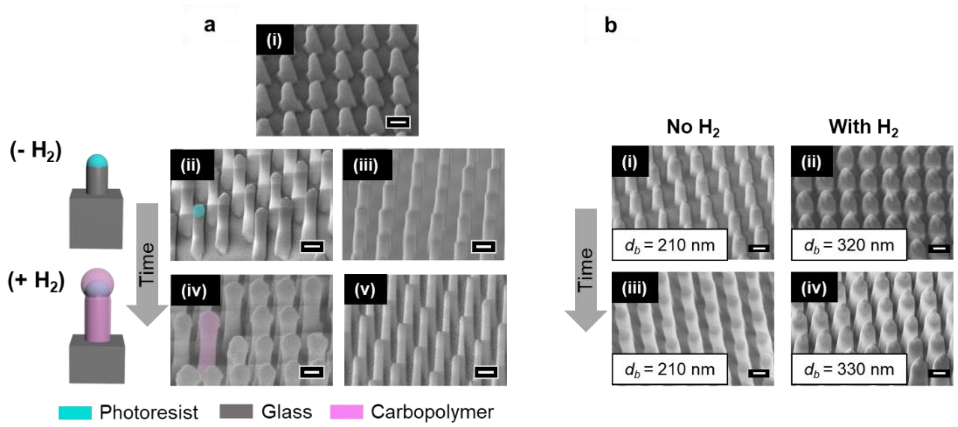

Figure S13 The effect of an RSML-mediated etch on nanostructure diameter, using a photoresist mask. a, 45°-tilted SEM images and schematics of structures generated after etching from photoresist mask (i) with p=350 nm, h=380 nm, d_b=230 nm. Before (left) and after (right) oxygen cleaning, of glass nanopillars etched in the (ii) absence and (iv) presence of H_2. The added color in SEM images indicates how the depleted mask in (ii), limiting further etching, can be regenerated by using the RSML concept (iv) to increase its durability, which permits deeper etching. **b,** The same mask is etched for an initial 7 min followed by a further 7 min in the absence (i,iii) or presence (ii, iv) of H_2. Without H_2 present, the diameter of the nanopillar is observed to slightly decrease with respect to that of the mask. In contrast, when H_2 is present, a significant increase in the diameter of the nanopillars is observed, and continues to increase with the etch time. Scale bars = 200 nm.

Nonetheless, even for tall photoresist masks, applying RSML at the beginning of the process has the benefit of tuning the diameter of the structure. In Figure S13b, we highlight this effect through comparison of the nanostructure evolution in the presence or absence of H_2. The SEM micrographs reveal that without H_2, a slight decrease (~10%) in the base diameter (d_b) is observed compared to that of the mask, however the d_b is maintained as etching proceeds from 7 to 14 mins, indicating anisotropic etching. Nevertheless, the thin remaining mask (~20 nm) indicates further etching is not possible under these conditions. In contrast, with the addition of H_2 = 6 sccm, a ~40% increase in d_b is observed after 7 min (in respect to the mask), which rises

by a further ~4% after a total of 14 min etching. This generates a tapered nanostructure profile, with a d_b (~330 nm) approaching the pitch (~350 nm). Overall, by interchanging the soft mask (BCP to photoresist), we demonstrate the versatility of RSML and highlight three scenarios where it can be employed: (i) to instantly increase the durability of a thin soft mask (*e.g.*, BCP), (ii) to re-build a depleted mask part-way through etching, and (iii) to tune the diameter and profile of the evolving nanostructure (originating from a thin or a thick mask).

Section S8. Mechanical stability

In order to test the mechanical stability of the nanopillars, we conducted tape-peel tests on two surfaces, differing in aspect ratio (AR) and solid fraction (f_s). Structures with larger a AR are known to have a lower mechanical stability[15] than those of smaller AR, therefore to compare, structures with an AR =2.5, and AR =5.5 were chosen. As the tape test was conducted without functionalizing the structures, it was anticipated that adhesion of the tape to the surface would be high owing to the larger contact area. Therefore, to further challenge the structures of lower AR, a sample with a high f_s was chosen (f_s = 0.5). The sample of higher AR, on the other hand, had a f_s = 0.1.

The (unfunctionalized) samples and a control (flat) sample were cleaned beforehand, and the tape (Scotch Magic ™ tape) was applied by hand and flattened onto the surface using the back of a pair of tweezers. The tape was subsequently peeled from one edge of the sample using tweezers. Removal of the tape from the low AR sample proved extremely challenging, with a very high adhesion causing the tape to tear. However eventual removal of the tape yielded a surface with a significant coverage of residual tape adhesive; which could be partially removed by sonication in acetone and IPA for ~5 min. The tape contacting the higher AR sample was less challenging to remove, and moreover the tape contacting the flat sample was easy to remove. Upon imaging of the samples under SEM, it was revealed the pillars remained intact, however large areas remained embedded within the adhesive.

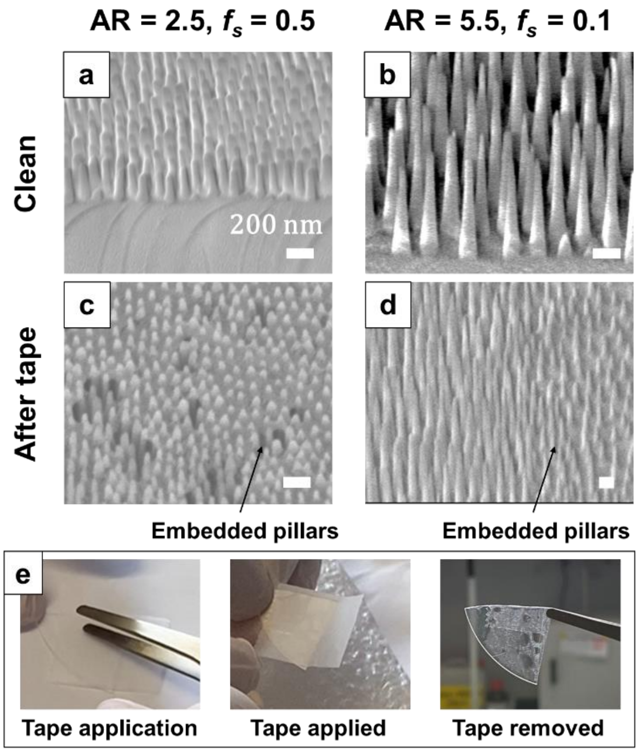

Figure S14 Tape-peel test. a,b, Two unfunctionalized surfaces with nanopillars of AR =2.5 and solid fraction fs 0.5 (a), and AR = 5.5 with fs 0.1 (b) were imaged via SEM prior to the tape peel test. **c,d,** After the tape-peel test, samples were re-imaged via SEM revealing intact pillars with regions embedded in residual adhesive from the tape. **e,** Photographs showing how the tape-peel test was performed. Scale bars in **a-d** are 200 nm.

Section S9. Optics

Optical Transmission Measurements

To calculate the absolute value of sample transmittance, a reference measurement was taken without a sample attached to the integrating sphere. A dark noise measurement was taken with the light source switched off. The device transmittance $T(\lambda)$, which is a function of wavelength λ, was calculated by $T(\lambda) = \frac{S_{sample}(\lambda) - S_{noise}(\lambda)}{S_{ref}(\lambda) - S_{noise}(\lambda)}$ where $S_{sample}(\lambda)$, $S_{ref}(\lambda)$, and $S_{noise}(\lambda)$ are the intensity counts measured by the spectrometer for the sample, reference (air) and dark noise respectively. The resultant calculated transmittances were normalized to the response of the eye (2008 CIE photopic luminous efficiency function).[16]

Optical Model

Nature has developed advanced antireflection strategies based on nanocone geometries with prominent examples found in the eyes of moths and the wings of butterflies.[17] The field of bioinspired antireflectance is well studied in the literature and commonly treated within the framework of effective medium theory (EMT).[18,19] According to the EMT picture, air and SiO_2 blend within the sub-wavelength nanostructure to produce a homogenous medium with a gradient refractive index profile in the axial *z*-direction, as shown in Figure S15a. The minimum wavelength λ_{min} for which reflectance is suppressed, is broadly considered to coincide with the limit where the first diffraction order of the grating transitions from evanescent to a real wave and depends linearly on the pitch p of the nanostructure array via $\lambda_{min} \sim p(n_{SiO_2} + sin\theta_i)$. Here, n_{SiO_2} is the refractive index of glass and θ_i is the angle of incidence.[15,20] The calculation of the maximum operating wavelength, λ_{max}, is more complicated and depends on the precise geometry of the pillars and the incident polarisation. However, it increases with the height h of the nanocones and as a rule of thumb it scales for conical structures as $\lambda_{max} \sim 2hcos\theta_i$.[21]

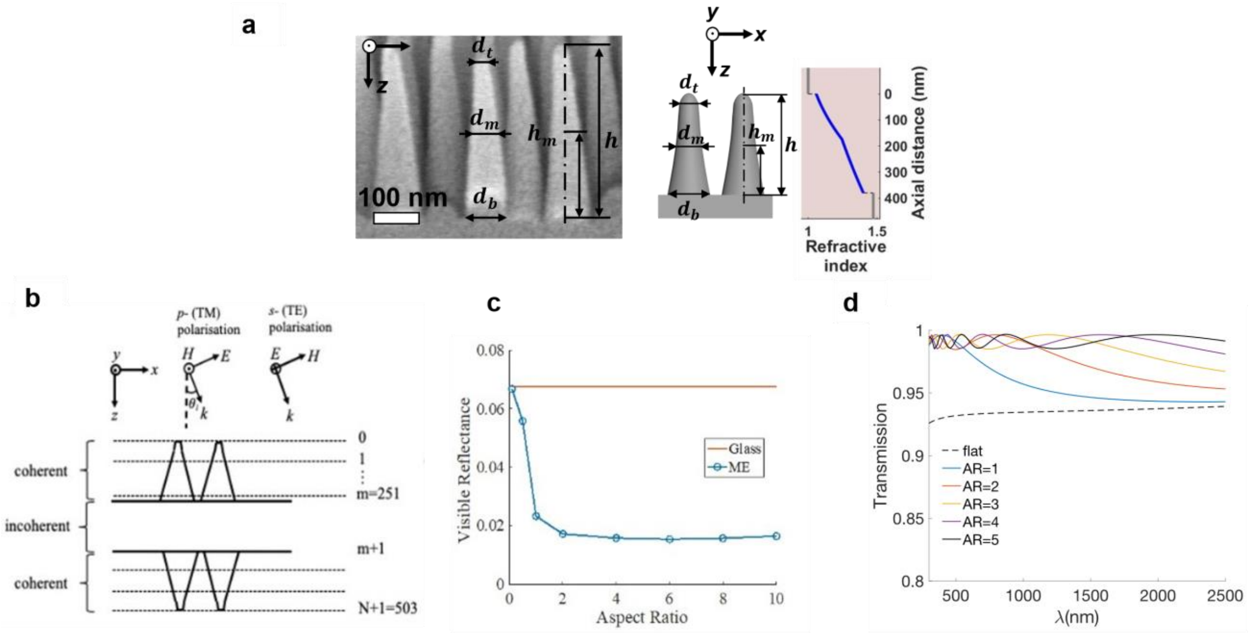

Figure S15 Optical Model. a, Nanocone geometry and gradient refractive index profile calculated by effective medium theory, where *d*, *h* are diameter and height, respectively; and *t*, *m*, *b* subscripts refer to the top, middle, and base, respectively. **b,** Transfer matrix method implementation in a multilayer coherent system. **c,** Simulated reflectance (calibrated to the human photopic response) for double-sided nanocones (blue line) and control glass sample for aspect ratios covering the range 0.1<AR<10. **d**, Simulated transmission as a function of wavelength for double-sided nanocones and control glass sample for aspect ratios covering the

range 0.1<AR<5. The subtle rippling in the transmission spectra is due to the imperfect refractive index matching at the bottom of the nanocones causing minute constructive and destructive effects.

To gain insight into the antireflection mechanism and to assess its tolerances when varying critical parameters, we developed a model by combining EMT with the coherent-incoherent transfer matrix method.[22,23] The double-sided structure to be analyzed consisted of two sets of nanocone arrays 380 nm tall each, separated by a thick (500 μm) fused silica substrate (see Table S1). The presence of the thick intermediate substrate rules out the usage of the traditional coherent transfer matrix method, where both the amplitude and phase of waves are considered, as this would result in a number of spurious Fabry-Perot types oscillations in the transmitted/reflected spectra, which are not observed experimentally (see Figure 4b in the main text). This is because the wide linewidth of the sources (LEDs, tungsten lamps, the Sun etc.), and the wide bandwidth of the detectors (CMOS cameras, human-eye) used in typical illumination systems, result in an averaging over the phases that eliminates any such fringes. In our implementation of the generalized matrix method, the nanocone regions were divided into a multilayer stack of 251 layers each and were treated with the coherent transfer matrix formulation as shown in Figure S14b. Increasing the number of layers did not result in further improvements in the accuracy of the simulations. On the other hand, the intermediate substrate was treated incoherently through its intensity matrix whereby interference effects due to phases accumulated from the round-trip passes through the thick substrate are ignored. This process resulted in smoothing out the high frequency oscillations in the simulated spectra, showing excellent agreement between experiments and theory as shown in Figure 4c in the main text. Modelling was conducted for both TE (*s*-) and TM (*p*-) polarizations with the two intensities being averaged incoherently at the end. To gain insight on the required ARs, we applied our model to simulate reflectance for double-sided nanocones with varying 0.1<AR<10 in Figure S14c. It can be clearly seen that AR>2 is sufficient to effectively suppress reflectance across the whole visible range which we verify experimentally in this work. We also extended our simulations into the NIR, Figure S15d, and estimated that for AR~5, high transmission is attained all the way to 2500 nm.

Section S10. Double-sided patterning

Nanostructuring on both sides of a glass substrate results in further enhanced light transmission across a broad range of wavelengths and incident angles. However, in terms of fabrication this is non-trivial, as it risks damaging the already patterned side, and furthermore the conditions of etching may be altered the second time round, meaning any deviations from the original recipe need to be accounted and compensated for. To minimize these issues, we developed the process shown in Figure S15a. Here, we first spin-coat a layer of photoresist PR on side 2 (S1818 MICROPOSIT™, 3k rpm for 35 s; bake at 115 °C for 1 min), which serves to not only protect the glass from scratches, but also minimize alterations in etching conditions, as it ensures both etching processes proceed with an insulating layer of PR on the opposite side, in contact with the platen. The viscosity of the PR should be carefully chosen to allow sufficient pattern coverage. Polyvinyl alcohol (PVA) could be an alternative solution as well as a resist without photosensitivity. Following this, side 1 is cleaned with an acetone wipe and oxygen plasma, and then the BCP is spin-coated. The subsequent RSM lithography process is performed as described in the main text, with an oxygen plasma at the end to remove carbopolymer deposition, and sonication bath with acetone (three times, 2 min) to remove the protective PR layer on side 1 (nanostructured side), PR is spin-coated onto the etched surface for protection, and then on side 2, the surface is cleaned with an acetone wipe and oxygen plasma, and is subsequently spin-coated with BCP and etched as before. The resulting surface (Figure S16b; Figure 4a), structured on both sides is sonicated in acetone to remove the remaining PR.

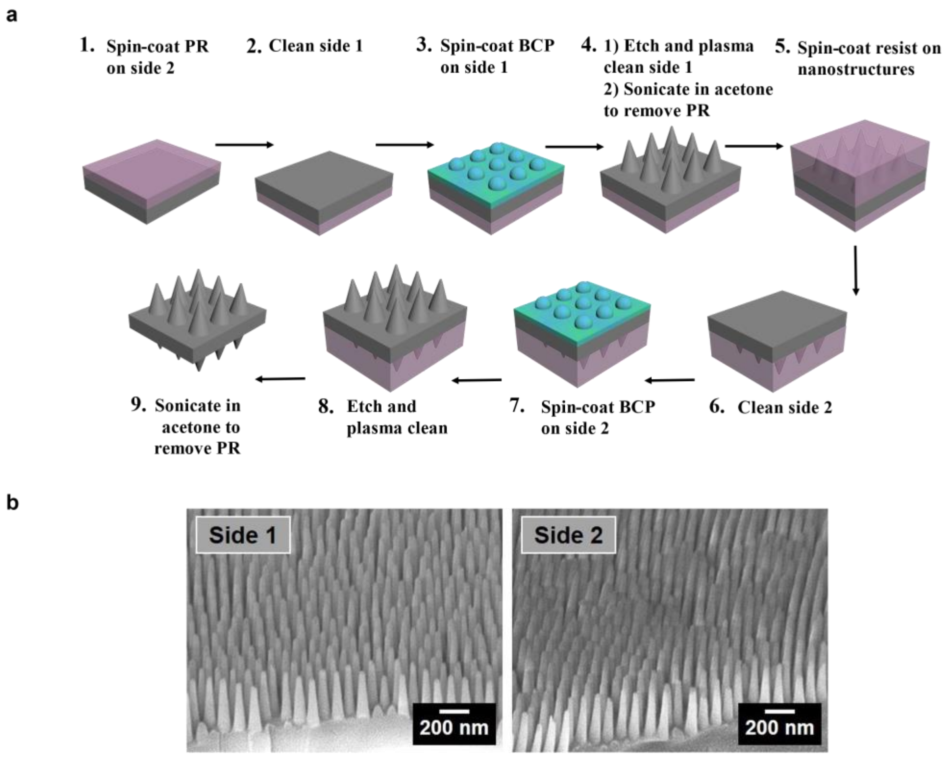

Figure S16 Double-sided glass processing. a, Schematic illustration of the double-sided fabrication process consisting of: (1) Spin-coating of a thin photoresist PR layer onto side 2. (2) Flipping of sample, and cleaning side 1 using acetone and a brief oxygen plasma. (3) Spin-coating of BCP micelles onto side 1. (4) Cyclic etching to generate nanostructures, with final oxygen plasma to remove deposition, and sonication in acetone to remove PR from side 2. (5) Spin-coating a layer of PR onto the fabricated nanostructures for protection. (6) Cleaning of side 2 by an acetone wipe and brief oxygen plasma. (7) Spin-coating of BCP micelles onto side 2. (8) Etching to generate nanostructures through our cyclic etching process, with oxygen plasma to remove deposition. (9) Sonication of the sample in acetone to remove PR layer. **b**, SEM image of the resulting double-sided sample used for modelling. The dimensions are listed in the Table S1 below.

Table S1. Critical nanocone parameters used in the optical model.

**Sample**	d_b	d_m	d_t	h	h_1	p
P100	92 nm	72 nm	35 nm	380 nm	206 nm	95 nm

The optical properties of this sample were further characterized (Figure 4) and the geometrical dimensions listed in Table S1 were used as an input for modelling. Additionally, we characterized the level of haze, by using an Imaging Sphere (Radiant Zemax). The instrument operates by Fourier transforming the far-field pattern of a beam transmitted through a transparent

sample, essentially resolving the angular distribution of the transmitted field. We took measurements of a beam profile after it passed through the sample (normal incidence), and corresponding reference measurement of the beam passing through air. By calculating the deconvolution of the two distributions, we were able to derive a scattering transfer function for the sample in the Fourier domain. Figure S17 shows the scattering transfer function of the sample as a function of angle for three representative visible wavelengths; 450 nm, 550 nm and 650 nm. As can be seen from the figure, the scattering transfer function appears as almost a delta function indicating that all energy is concentrated at $\theta = 0°$ (measured from the normal to the sample) and so, we can see no scattering present within the resolution of our instrument (0.5 degrees).

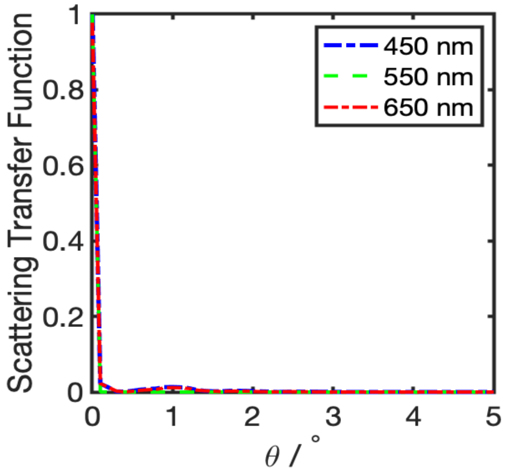

Figure S17 Haze measurements. Scattering transfer function, as a function of angle θ, for the double-sided sample for measurements at 450 nm (blue), 550 nm (green) and 650 nm (red).

Section S11. Wettability

Many factors contribute to the wettability of a surface, for instance a low solid fraction, high roughness, structural homogeneity, and inherent hydrophobicity. Through our process, and subsequent functionalization with a hydrophobic silane, these are addressed, and superhydrophobicity is successfully obtained. However, to further augment superhydrophobicity, we perform a brief post-processing modification with diluted hydrofluoric acid (HF, 5%), which acts to reduce the solid fraction $\emptyset_s$, calculated for a hexagonal array as: $\emptyset_s = \frac{2\pi r^2}{p^2\sqrt{3}}$, where r = radius of curvature of the tip, p = pitch. Physically, the isotropic etching by HF sharpens the tips,

whilst also reducing the height and diameter, depending on the process time (SEM images; Figure S17). The effect on wettability becomes clear when comparing the advancing contact angle θ_{adv} of P100 and P400 samples, with and without sharpening as seen in Figure S18. There, θ_{adv} is plotted as a function of *r*/*p*, alongside values predicted by the Cassie-Baxter equation: $\cos\theta_{CB} = \emptyset_s(\cos\theta_E + 1) - 1$, where θ_E is the advancing contact angle on the corresponding smooth surface.

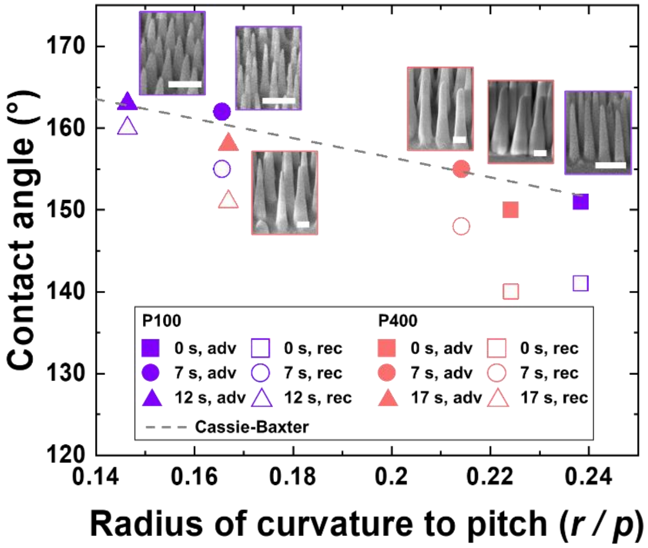

Figure S18 The effect of sharpening on the sample wettability. Plot of the advancing and receding contact angles as a function of the radius of curvature to the pitch (*r* / *p*) with corresponding SEM images for the P400 (pink) and P100 (purple) samples with and without HF sharpening (0, 7 and 17s for P400, 0, 7 and 12s for P100). Plotted alongside is the Cassie-Baxter predicted trendline (grey dashed). Scale bars = 200 nm.

The coefficient of restitution of droplet bouncing ε also provides a good indication of the global hydrophobicity. It highlights surface inhomogeneities manifested as pinning points which result in a low elasticity. The droplet bouncing of the sharpened P100 with a θ^{a}_{adv}=163° was probed as a function of time (Figure S19) by releasing a droplet of *r* = 0.9 mm onto the sample from a height of 1 cm. This gave rise to an initial velocity *V* = 0.33 m/s and *We* =1.33, where *We* is the dimensionless Weber number which is a ratio between deforming inertial forces and stabilizing cohesive forces of a fluid. The coefficient ε can be inferred after each bounce using the following formula ε=*V'*/*V*, where *V'* and *V* are the velocities after and before the impact, respectively. At high velocity (first bounce), a modest elasticity is observed with ε = 0.5, however, the now decreased velocity of the second bounce (*V*=0.13 m/s, *We* = 0.2) results in much greater

elasticity, with ε = 0.90. This indicates the presence of a threshold velocity for bouncing, with greater velocities providing a larger degree of deformation and vibration (energy loss). Remarkably, the drop undergoes 17 bounces before coming to rest, with no further detachment from the surface.

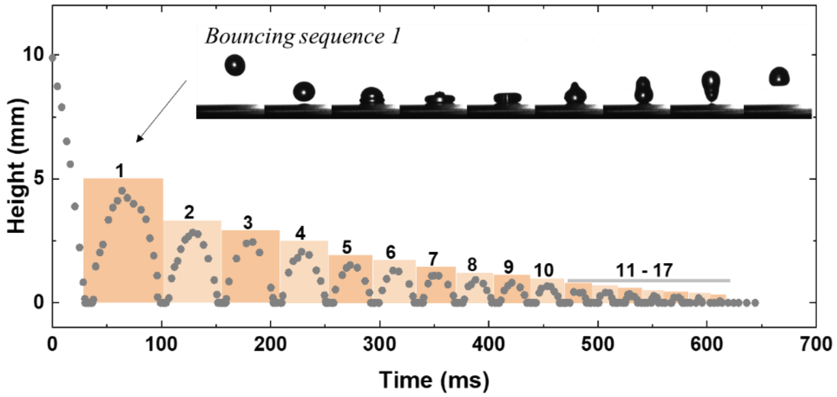

Figure S19 Rebound characterization of a water droplet. Rebound height of a 3μL droplet on surface P100 vs time. The droplet was released from an initial height of 1cm, and bounces a remarkable 17 times. The inset shows an example bounce.

The study into droplet impact dynamics was carried out using a high-speed camera (retraction with bouncing or not) at increasing velocities V = 0.4-4.4 m/s. Note, above V=2 m/s, formation of satellite droplets occurs, preventing the measurement of droplet diameter D. Whilst no difference was observed between samples during droplet expansion, the retraction process did differ; whereby the droplet on P400 took longer to retract than on P100. For instance, at V = 1.0 m/s, we observe a contact time τ =13.3 and 16.3 ms for P100 and P400, respectively. At the higher velocity of 2.0 m/s though, the effect becomes more pronounced with τ=13.9 and 19.3 ms. As P100 comprises a higher density of structures, one might expect the opposite trend since a greater area would be wetted by the droplet. However, the resistive capillary pressure is expected to be higher for P100 compared to P400 due to the reduced pitch, thus giving rise to a lower wetted area.[24]To estimate the penetration depth of water into the nanostructure, it is necessary to consider the pressures acting on the surface by an impacting water droplet:[15,25]

(i) Water hammer pressure, which is the pressure surge caused when the moving droplet hits the surface forcing a momentum change. It is calculated to be 0.6 MPa at a velocity of 2.0 m/s according to:[26]

$$P_{WH} \approx K_{WH} \rho c V_0$$

Where K_{WH} is the water hammer pressure coefficient (typically varies from 0.001-0.2), ρ is the density of water, c is the speed of sound in water, and V_0 is the impacting velocity of the droplet.

(ii) Simultaneously, pressure due to liquid compressibility is experienced as the droplet spreads, and is described by the Bernoulli equation to give a value of 2 kPa (supposing the velocity is still 2.0 m/s), according to:[26,27]

$$P_B = {}^{\rho}/_{2} V_0^2$$

Comparing P_{WH} and P_B with the critical pressure required to force the liquid meniscus into the nanostructure, P_c, indicates whether the liquid penetrates (P_{WH} or $P_B > P_c$), or not (P_{WH} or $P_B < P_c$). P_c is obtained through:

$$P_c = \frac{4\pi\gamma r cos(\theta-\alpha)}{\sqrt{3}p^2-2\pi r^2}$$

Where r and p are the radius and the pitch of the structure, θ is the advancing angle on the corresponding smooth surfaces, α is the cone angle, and γ is the interfacial tension of water (7.2×10^{-2} N m^{-1}).

The graph in Figure 4e shows how the critical pressure varies with penetration percentage (*z/h*; penetration depth over nanocone height), calculated through estimation of *r*, at different depth *z*. Similarly, the effects of aspect ratio can be seen in Figure S20. As the AR increases from 1 to 5, the resistive capillary pressure also increases. This is a result of the greater degree of unfavourable contact between the water droplet and the nanocones for an AR of 5 when compared to an AR of 1 at the same penetration depth (z/H).

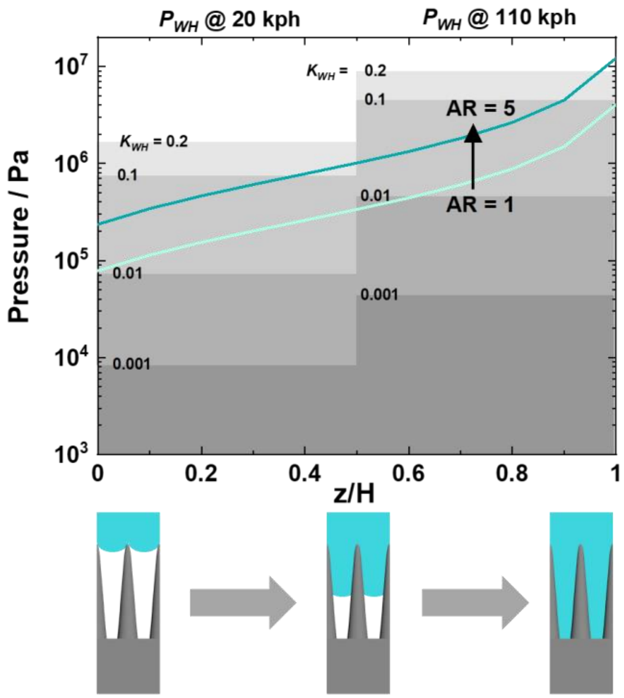

Figure S20 Capillary pressure versus penetration percentage. Calculated capillary pressure P_c plotted for nanocones of aspect ratio 1 and 5, as a function of the penetration percentage (*z/h*), where *z* is the depth of meniscus penetration and *h* is the total height. The water hammer pressure generated at impacts of 20 and 110 kph are marked on the graph, with varying values for the water hammer pressure coefficient K_{WH}. The bottom row of schematics serves as a representation of the penetration depth *z/h*.

Section S12. Model of interactions between nanopillars and bacteria cells

Unlike other approaches,[28,29] treatment of the interfacial energy gradient as the *driving force* promoting bacterial migration into the nanostructure, addresses the system in less abstract terms (energy as opposed to force).[30] The calculation of the interfacial energy gradient between cells and nanopillars follows the concept recently presented by Liu *at al.*,[30] with some adaptations. We focus here on the interactions with *S. aureus* possessing a volume of ~10^{-19} m^3, which was not considered in the reported study. Additionally, we investigate a hexagonally-packed array as opposed to square-packed[30] which results in an increase of surface roughness as well as solid fraction that is in contact with the cell.

Figure S21 illustrates schematically *S. aureus* cells, adhered to the flat (a) and structured surface with nanopillars (b). The pillars are characterized by pitch *p*, height *h*, and diameter *d*. Although *S. aureus* is considered a coccus, based on the SEM image processing, we treat its shape as a

non-perfect sphere with a short segment L between two hemispheres. After cell adhesion to the surface, a flat base is formed of the length L and width $2r_s$. The attached cell forms an adhesion angle with flat surface and nanopillars, θ_c and θ, respectively.

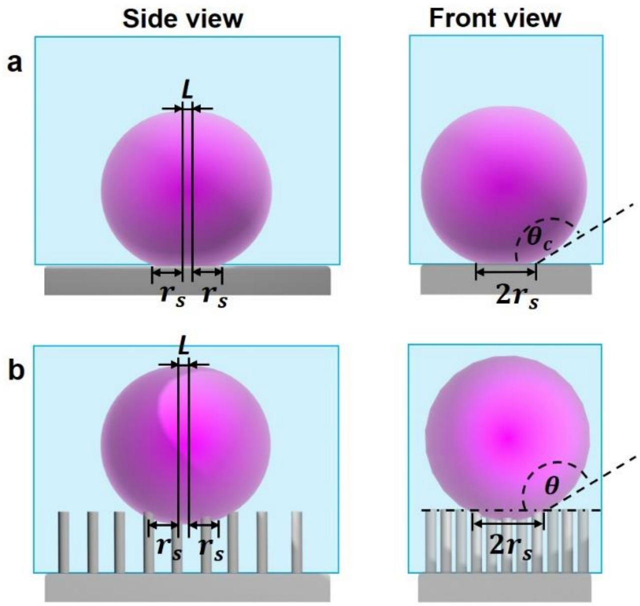

Figure S21 Schematic of *S. aureus* adhered to the surface. a, The cell on flat surface is in contact with the substrate *via* flat base of the length L and width $2r_s$, and an adhesion angle $\boldsymbol{\theta_c}$. The three interfaces are observed between the cell (purple), solid (grey) and liquid (blue) **b,** The cell on nanostructured surface is in contact with the substrate *via* flat base of the length L and width $2r_s$, and an adhesion angle $\boldsymbol{\theta}$. In this study, $\boldsymbol{\theta_c = 150°}$ and $\boldsymbol{\theta = 157°}$, as measured by ImageJ.

In this model, the interfacial energy gradient which involves nanostructures and the attached cells is considered as the driving force F_d of bacteria into the nanostructure and it includes: (i) Gibbs free surface energy γ and (ii) deformation surface energy σ (which concerns all the changes related to when the cell wall deforms). For a hydrophilic nanostructured surface, there are solely liquid and solid interfaces beneath cell. Hence, the energy E can be expressed as follows:

$$E = A_{up}\sigma_{CL} + A_{bCL}\sigma_{CL} + \left[(r - f)A_{total} - \left(A_{bCS} - fA_{pCS}\right)\right]\gamma_{SL} + f\left(A_{total} - A_{pCS}\right)\gamma_{SL} + A_{bCS}\sigma_{CS}$$

Where A_{up} – upper surface area of an adhered cell above the solid; A_{pCS} – adhered projection area between the cell and the solid surface; A_{total} – surface projection area including the cell; γ_{SL} – surface free energy of solid-liquid interface; σ_{CL} and σ_{CS} – deformation surface energy

between cell and liquid or solid, respectively; r – Wenzel roughness; and f – solid fraction that is in contact with the cell.

The gradient E is derived in this model like for the case when droplet migrates towards the nanostructures, and can be written as follows:

$$F_d = -\frac{dE}{dx} = -(\frac{\partial E}{\partial x} + \frac{\partial E}{\partial \theta}\frac{\partial \theta}{\partial x})$$

Where $\frac{\partial E}{\partial x}$ corresponds to changing rate of the interfacial energy when cell moves towards the structure along x, whereas $\frac{\partial E}{\partial \theta}$ refers to changes when adhesion contact angle varies. Ultimately, the force can be expressed as:

$$F_d =$$
$$\frac{r-1}{h}(\pi r_s^2 + 2r_s L)(\gamma_{SL} - \sigma_{CS}) +$$
$$2(\pi r_s + 2L)(1-f)\sin\theta\,\sigma_{CL}\frac{sin\theta(1-cos\theta)\pi r_s^2 + (sin\theta - \theta cos\theta)(1+cos\theta)r_s L}{sin\theta(1-cos\theta)\pi r_s^2 + 2(sin\theta - \theta cos\theta)(1+cos\theta)r_s L}$$

We calculate this force by using the following expressions for interfacial tension and deformation energies:

$$\gamma_{SL} = \frac{\gamma_{LG}}{2}(\sqrt{1+sin^2\theta_w} - cos\theta_w)$$

$$\sigma_{CS} = \frac{\sigma_{CL}}{2}(\sqrt{1+sin^2\theta_C} - cos\theta_C)$$

$$\gamma_{SL} = \frac{\sigma_{CL}}{2}(\sqrt{1+sin^2\theta_C} + cos\theta_C)$$

Where θ_w is an intrinsic water contact angle of material substrate (here, PBS on fused silica, θ_w=70°).

Additionally, solid fraction and roughness for hexagonally-packed pattern are given by:

$$f = \frac{2\pi(\frac{d}{2})^2}{p^2\sqrt{3}}$$

And

$$r = 1 + \frac{2\pi dh}{p^2\sqrt{3}}$$

Having the force calculated, we can obtain the value of the pressure P which is exerted on cells by nanopillars using the following equation:

$$P = \frac{F_d}{(\pi r_s^2 + 2r_s L)f}$$

Figure S22 presents the results of the pressure and driving force calculated for the topographies that vary in pitch and diameter in a range of 85-300 nm and 10-60 nm, respectively. The data indicate that indeed, the smaller the diameter (sharper the pillar) at the same pitch, the more pressure is exerted on the cells. Besides, the larger the pitch, the more pressure is generated. However, pitches as small as ~90 nm can be efficient as long as the diameter is small enough. Additionally, if that pressure is greater than the critical elastic stress of the cell wall (1 MPa in this case),[30] creep deformation occurs and depending on the deformation depth with respect to the height of nanopillars, penetration is possible or not.

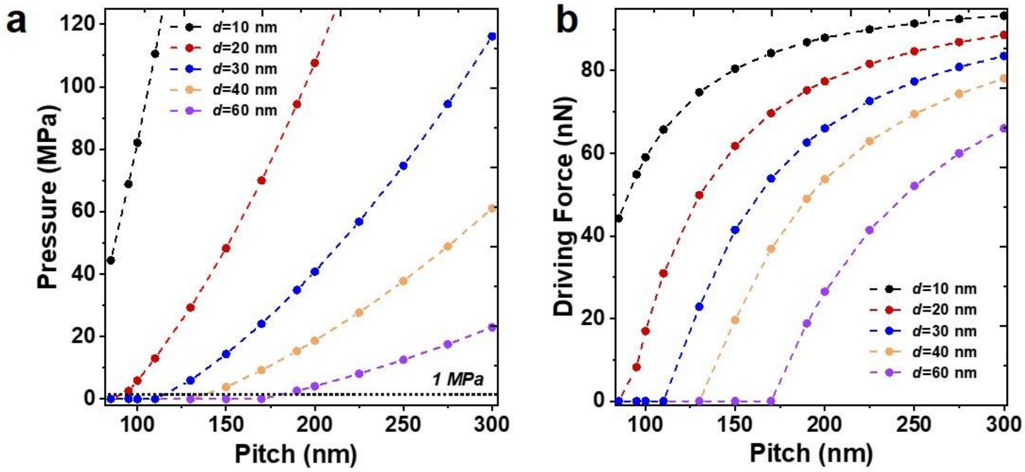

Figure S22 Effect of geometry of nanopillars on the interactions with *S. aureus*. a, The calculated pressure acting on the cell as a function of pitch, with varied diameters (10-60 nm). **b,** The calculated force acting on the cell as a function of pitch.

The calculated pressure for the case of p = 110 nm and d = 21±4 nm is ~10 MPa which indicates that creep deformation can take place. Furthermore, the SEM image analysis revealed numerous cells which were clearly ruptured, with some pillars penetrating the cells. This further suggests that h=230 nm was sufficient for cell penetration to occur. The calculated F_d is ~30 nN. It should be noted that considering the distribution of calculated diameter, the pressure value ranges from 3 to 24 MPa, thus still being greater than the critical elastic stress of the cell wall. Recently, it has been reported that a force of ~20 nN is required to exert critical damage to *Escherichia (E.) coli*

cells, measured by means of AFM and using a silicon tip with a nominal tip radius of 8-10 nm.[31] Since the force is greater than ~1 nN – typically used in the studies on creep deformation of bacteria cells which does not lead to cell puncturing,[32] the obtained value of ~20 nN seems appropriate. *E. coli* is a Gram-negative bacterium which possesses a thinner cell wall than Gram-positive bacteria like *S. aureus*, thus it is expected that a larger force is required in order to puncture the latter. Given that our average calculated force is greater (~30 nN) as well as the range of the generated pressures, the values seem reasonable.

Additionally, it is noteworthy that the model approximates the system of interactions and does not consider a contribution of kinetic energy that can enhance the force if motility is present, for instance. However, it does not concern our case as *S. aureus* is non-motile. Another important aspect though, which is not accounted for, refers to material flexibility. The energy storage-release mechanism has been proposed by Ivanova,[33,34] based first on carbon nanotubes and more recently, square-packed silicon nanopillars of 92 nm pitch. Broadly, it states that increasing pillar flexibility (through increasing the height), induces a higher degree of bacterial killing. The rationale being; pillars with greater flexibility are more susceptible to external forces, thus deflect towards the cell sitting atop the structure (storing energy), and it is during the subsequent release of mechanical energy as pillars return to their original locations, that the cell ruptures. However, the deflection is governed by the elasticity of the structures which deviates with material and shape, making it a challenge to predict for our hexagonally-packed glass cones. Although the extent of this effect is not clear in our case, nor its impact on the calculated pressure, we evidence that bending of the nanopillars beneath *S. aureus* cells indeed occurs, as shown in Figure S23.

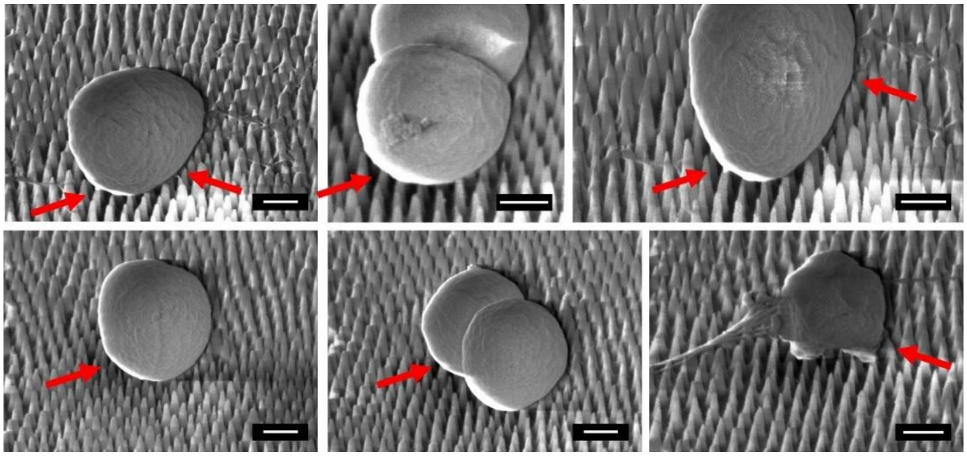

Figure S23 *S. aureus* cells attached onto glass nanopillars. SEM images of cells resting on the nanopillars with red arrows indicating the bending pillars. Scale bars 200 nm.

References:

[1] L. Wang, T. J. McCarthy, *Angew. Chemie Int. Ed.* **2016**, *55*, 244.

[2] T. Li, Z. Wang, L. Schulte, O. Hansen, S. Ndoni, *RSC Adv.* **2015**, *5*, 102619.

[3] K. W. Gotrik, A. F. Hannon, J. G. Son, B. Keller, A. Alexander-Katz, C. A. Ross, *ACS Nano* **2012**, *6*, 8052.

[4] C. Sinturel, M. Vayer, M. Morris, M. A. Hillmyer, *Macromolecules* **2013**, *46*, 5399.

[5] C. T. Black, K. W. Guarini, R. Ruiz, E. M. Sikorski, I. V. Babich, R. L. Sandstrom, Y. Zhang, *Tech. Dig. - Int. Electron Devices Meet. IEDM* **2006**, *51*, 605.

[6] S. Krishnamoorthy, Y. Gerbig, C. Hibert, R. Pugin, C. Hinderling, J. Brugger, H. Heinzelmann, *Nanotechnology* **2008**, *19*, 285301.

[7] S. Krishnamoorthy, R. Pugin, J. Brugger, H. Heinzelmann, C. Hinderling, *Adv. Funct. Mater.* **2006**, *16*, 1469.

[8] K. Mohamed, M. M. Alkaisi, *Nanotechnology* **2013**, *24*, 015302.

[9] C.-M. Chan, T.-M. Ko, H. Hiraoka, *Surf. Sci. Rep.* **1996**, *24*, 1.

[10] F. Marty, L. Rousseau, B. Saadany, B. Mercier, O. Français, Y. Mita, T. Bourouina, *Microelectronics J.* **2005**, *36*, 673.

[11] S. Huang, S. Shim, S. K. Nam, M. J. Kushner, *J. Vac. Sci. Technol. A* **2020**, *38*, 023001.

[12] S.-W. Cho, J.-H. Kim, S. Kim, E. W. Shin, C.-K. Kim, *ECS J. Solid State Sci. Technol.* **2015**, *4*, P226.

[13] J. K. Kim, S. H. Lee, S. Il Cho, G. Y. Yeom, *J. Vac. Sci. Technol. A Vacuum, Surfaces, Film.* **2015**, *33*, 021303.

[14] M. K. Choi, H. Yoon, K. Lee, K. Shin, *Langmuir* **2011**, *27*, 2132.

[15] K.-C. Park, H. J. Choi, C.-H. Chang, R. E. Cohen, G. H. McKinley, G. Barbastathis, *ACS Nano* **2012**, *6*, 3789.

[16] "UCL colour and vision database," **n.d.**

[17] P. B. CLAPHAM, M. C. HUTLEY, *Nature* **1973**, *244*, 281.

[18] R. H. Siddique, G. Gomard, H. Hölscher, *Nat. Commun.* **2015**, *6*, 6909.

[19] O. Deparis, N. Khuzayim, A. Parker, J. Vigneron, *Phys. Rev. E* **2009**, *79*, 041910.

[20] I. H. Malitson, *J. Opt. Soc. Am.* **1965**, *55*, 1205.

[21] E. B. Grann, M. G. Moharam, D. A. Pommet, *J. Opt. Soc. Am. A* **1995**, *12*, 333.

[22] C. L. Mitsas, D. I. Siapkas, *Appl. Opt.* **1995**, *34*, 1678.

[23] C. C. Katsidis, D. I. Siapkas, *Appl. Opt.* **2002**, *41*, 3978.

[24] T. Maitra, M. K. Tiwari, C. Antonini, P. Schoch, S. Jung, P. Eberle, D. Poulikakos, *Nano Lett.* **2014**, *14*, 172.

[25] A. Checco, A. Rahman, C. T. Black, *Adv. Mater.* **2014**, *26*, 886.

[26] T. Deng, K. K. Varanasi, M. Hsu, N. Bhate, C. Keimel, J. Stein, M. Blohm, *Appl. Phys. Lett.* **2009**, *94*, 133109.

[27] M. Reyssat, A. Pépin, F. Marty, Y. Chen, D. Quéré, *Europhys. Lett.* **2006**, *74*, 306.

[28] S. Pogodin, J. Hasan, V. A. Baulin, H. K. Webb, V. K. Truong, T. H. Phong Nguyen, V. Boshkovikj, C. J. Fluke, G. S. Watson, J. A. Watson, R. J. Crawford, E. P. Ivanova, *Biophys. J.* **2013**, *104*, 835.

[29] F. Xue, J. Liu, L. Guo, L. Zhang, Q. Li, *J. Theor. Biol.* **2015**, *385*, 1.

[30] T. Liu, Q. Cui, Q. Wu, X. Li, K. Song, D. Ge, S. Guan, *J. Phys. Chem. B* **2019**, *123,* 8686.

[31] A. del Valle, J. Torra, P. Bondia, C. M. Tone, P. Pedraz, V. Vadillo-Rodriguez, C. Flors, *ACS Appl. Mater. Interfaces* **2020**, *12*, 31235.

[32] Y. Bu, L. Li, J. Wang, *Sci. China Technol. Sci.* **2019**, *62*, 781.

[33] D. P. Linklater, M. De Volder, V. A. Baulin, M. Werner, S. Jessl, M. Golozar, L. Maggini, S. Rubanov, E. Hanssen, S. Juodkazis, E. P. Ivanova, *ACS Nano* **2018**, *12*, 6657.

[34] E. P. Ivanova, D. P. Linklater, M. Werner, V. A. Baulin, X. Xu, N. Vrancken, S. Rubanov, E. Hanssen, J. Wandiyanto, V. K. Truong, A. Elbourne, S. Maclaughlin, S. Juodkazis, R. J. Crawford, *Proc. Natl. Acad. Sci.* **2020**, *117*, 12598.

Supporting Videos

Video S1 – Regenerative Secondary Mask Lithography (RSML) process.

Video S2 – Slow motion video of a water stream upon impacting the (i) nanostructured and (ii) flat glass. The glass surface is tilted at 45°. Note how the water droplets bounce off the structured surface, whereas on the flat, they spread.

Video S3 – Slow motion video of a continuous stream of droplets deposited onto nanostructured glass (P100). The continuous stream of water emulates real-world conditions, thus indicating the structured surface performs under these conditions.

Video S4 – Slow-motion video of a bouncing droplet upon impacting the nanostructured glass (P100). The video was recorded using a high-speed camera. A water droplet of r = 0.9 mm was deposited from a height of 1 cm, impacting the surface with initial velocity V = 0.33 m/s, giving rise to 17 bounces. This indicates the excellent global superhydrophobicity, and lack of pinning – an attribute to the sample uniformity.

Video S5 – Slow-motion video of a high-speed droplet impact on the nanostructured glass (P100). Video was recorded using a high-speed camera. The droplet of r = 1.35 mm was deposited from a height of 1m, giving rise to a velocity V = 4.4 m/s. Satellite droplets form upon impact and the droplet spreads beyond the width of the sample, however after retraction no pinning can be observed.
